# Supplementary material for: Resolving nanostructure and chemistry of solid-electrolyte interphase on lithium anodes by depth-sensitive plasmon-enhanced Raman spectroscopy
Source: Nat Commun. 2023 Jun 15;14:3536. doi: 10.1038/s41467-023-39192-z (PMC10272176; doi:10.1038/s41467-023-39192-z)
Supplement: Supplementary file 1 — Supplementary Information [file 41467_2023_39192_MOESM1_ESM.pdf]

Supplementary Information

Resolving nanostructure and chemistry of solid-electrolyte interphase on lithium anodes by depth-sensitive plasmon-enhanced Raman spectroscopy

Yu Gu[1,#], En-Ming You[1,#], Jian-De Lin[1], Jun-Hao Wang[1], Si-Heng Luo[1], Ru-Yu Zhou[1], Chen-Jie Zhang[2], Jian-Lin Yao[2], Hui-Yang Li[1], Gen Li[1], Wei-Wei Wang[1], Yu Qiao[1], Jia-Wei Yan[1], De-Yin Wu[1], Guo-Kun Liu[3], Li Zhang[1], Jian-Feng Li[1], Rong Xu[4], Zhong-Qun Tian[1]*, Yi Cui[4]*, Bing-Wei Mao[1]*

[1]State Key Laboratory of Physical Chemistry of Solid Surfaces, iChEM, Innovation Laboratory for Sciences and Technologies of Energy Materials of Fujian Province (IKKEM), College of Chemistry and Chemical Engineering, Xiamen University, Xiamen, China.

[2]College of Chemistry, Chemical Engineering and Materials Science, Soochow University, Suzhou, China.

[3]State Key Laboratory of Marine Environmental Science, College of the Environment and Ecology, Xiamen University, Xiamen, China.

[4]Department of Materials Science and Engineering, Stanford University, Stanford, CA, USA.

[#]These authors contributed equally.

*e-mail: bwmao@xmu.edu.cn; yicui@stanford.edu; zqtian@xmu.edu.cn

This file includes:

Supplementary Notes 1–6

Supplementary Figures 1–28

Supplementary Tables 1–5

Supplementary References

Supplementary Notes

S1. The limitations of individual SERS/SHINERS for studying SEIs

SERS is a powerful technique for in situ characterization of interfacial processes with molecular-level fingerprint information in a large spectral range including low wavenumber region. Due to the necessity of signal enhancement, previous SERS investigations are mostly limited to systems that involve substrates bearing strong LSPs such as nanostructured Au and Ag which are however not common anode materials for batteries. Although Li metal has s-electron shell and is also expected to exhibit LSPs necessary for studying of Li anode, only one previous in situ study has been reported by authors' group[1]; since the work was carried out in a specific electrolyte containing trace amount of H_2O as additive to create nanostructured Li during deposition for generating LSPs, the obtained information is somehow oversimplified, which limited its universal significance. On the other hand, in the anode-free configuration of Li anodes, Cu as a standard material for current collector is a coinage-metal that can excite LSPs and could in principle be adopted to enhance SEI signals without the need of foreign SERS-active metals; but such studies have not been reported up to now.

Alternatively, SHINERS using plasmonic metal cores coated with ultrathin inert shells that separate cores from the substrate are used solely as Raman signal amplifiers, not only breaks the limitation of SERS substrate generality, but also guarantee reliable signals from interfaces. However, works on SEIs of Li metal anodes using SHINERS have been rarely reported, probably due to complex operation procedures for SHINERS measurements on extremely reactive Li metal surfaces and employment of organic electrolytes which require special concerns in various aspects.

No matter which situation, SERS or SHINERS, each individual LSPR enhancement strategy could only provide information of SEIs in the very vicinity of the corresponding LSPR-active source, given that the exponential decay of electromagnetic field enhancement within a couple of nanometers, while the SEI is tens of nanometers thick with vertical distribution of components.

S2. The advantage of nanostructured Cu over flat Cu in integrated Cu-SHINs substrate for studying SEIs by Raman spectroscopy

SEI components usually have low Raman scattering sections and have to rely on SERS. However, since SERS activity is strongly nanostructure dependent, it is extremely difficult, if not impossible, to directly use flat Cu substrate that cannot support effectively strong LSPs to

study SEIs. Whereas, this situation can be circumvented by borrowing LSP-active SHINs introduced on flat Cu surface to enhance the Raman signals of SEIs grown on Cu surface. For this system, there are only two types of hotspots located at the junctions of flat Cu-to-SHINs and SHIN-to-SHIN that in principle enhance the Raman signals of SEI in the inner and outer region, respectively. The measured bands associated with SEI components are highly complex and dynamically varying, especially at the initial stage of SEI formation. Therefore, to obtain higher detection sensitivity for SEIs, the flat Cu with SHINs is replaced by the SERS-active nanostructured Cu created by electrochemical ORC method. Three different type of hotspots can be formed in the junctions of Cu-to-Cu (I), Cu-to-SHIN (II) and SHIN-to-SHIN (III), respectively, to form a more active integrated plasmonic enhancement substrate with unique depth sensitivity. In the early stage of SEI formation, the hotspots (I) and (II) enhance the Raman signals of SEI in the inner region. During the growth of SEI, hotspots (II) and (III) act to detect the chemical structure of SEIs including outer regions. In this circumstance, the plasmonic coupling is strengthened and further enhanced Raman signals of SEI can be detected. It is noted that the application of 3D current collector is one of the conventional strategies to improve the performances of Li metal anodes[2–4]. And the nanostructured Cu surface with slightly increased surface area as well as inhomogeneous charge distribution does not seem to significantly affect the SEI chemistry, at least on a macroscopic scale, as evidenced by similar CVs between nanostructured and plain Cu surfaces (Supplementary Fig. 10) and similar spectroscopic features observed in different regions of nanostructured Cu-SHINs substrate with and without continuous laser irradiation during SEI formation process (figure shown below). Overall, the advantage of such combination of nanostructured Cu and SHINs lies in the synergistic plasmonic enhancement effect, which provides much higher detection sensitivity of Raman signals from SEIs and related interfaces with depth sensitivity, enabling bottom-up monitoring of the vertical evolution of SEI during different formation stages.

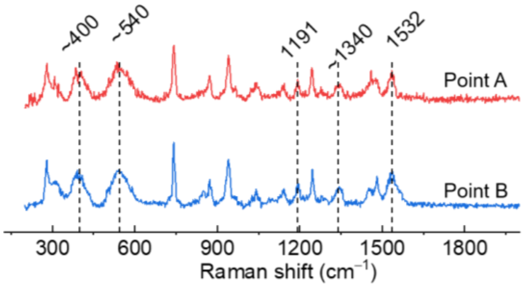

Supplementary Figure | Comparison of Raman spectra recorded in different regions of Cu-SHINs substrate at 1.0 V (vs. Li/Li+) in DOL-based electrolyte. Point A: SEI formation under continuous laser irradiation; Point B: SEI formation without continuous laser irradiation.

S3. The finite-element method simulation of optical electric-field distribution at initial integrated Cu-SHINs substrate

The electric field distribution and enhancement from initial integrated Cu-SHINs substrate was modelled using a finite-element method (FEM). The structure selected for modelling was described in Supplementary Methods. Due to the potentially broad size distribution of the nanostructured Cu surface obtained by electrochemical ORC method, we have modelled the electric-field distribution for the integrated Cu-SHINs substrate with both larger and smaller Cu islands. As illustrated in Supplementary Fig. 3, in both cases, the regions of enhanced field strength (i.e. hotspots) are formed under laser excitation in three types of junctions, namely Cu island-to-Cu island, Cu island-to-SHIN and SHIN-to-SHIN, and their average plasmon enhancement factors (EFs) exceed 8 orders of magnitude (with 785 nm laser), which is theoretically strong enough for ultrasensitive detection of SEI components. In principle, in the early stages of SEI formation, the hotspots located in the junctions of Cu-to-Cu and Cu-to-SHIN enhance the Raman signals of SEI in the inner region. During the growth of SEI, hotspots located in the junctions of Cu-to-SHIN and SHIN-to-SHIN act to detect the chemical structure of SEIs including outer regions. This simulation indicates that the integrated Cu-SHINs substrate with multi-hotspots can serve as a favorable surface plasmon-enhancement system for bottom-up probing the formation and evolution of SEIs in real-time even with the presence of the background electrolyte.

S4. The influence of SEI growth on the electric-field distribution in the integrated Cu-SHINs substrate

In the case of traditional solid-liquid interface, it is relatively easy to perform SERS/SHINERS analysis of general adsorbates in the well-controlled nanogap between nanoparticle and substrate surface, since their position on the substrate remain largely constant during the electrochemical processes. However, the situation is totally different in the solid-solid interface such as the metal-SEI interface under investigation in this work, in which SEI grows in vertical direction and changes dynamically, thereby affecting the local electromagnetic field distribution and the plasmon enhancement performance of the integrated Cu-SHINs substrate. It is noteworthy that the presence of SHINs introduces a shielding effect for electrolyte reduction and thus SEI growth in the gap between SHINs and metal surface. Therefore, SEI formation is initiated only at the metal surface, and the growth of SEI is expected to leave the SHINs partially embedded in the SEI (Supplementary Fig. 4). Due to the fact that Raman signals decrease exponentially with increasing distance, the possibility of SEI growth in the gap between SHINs

and metal surface (i.e. beneath the SHINs so that SHINs float on the SEI) can be excluded because under such a circumstance Raman signals of SEI components could not be detected efficiently, which is contradictory to our experimental results.

To investigate the influence of SEI growth on the electromagnetic field distribution, FEM simulations were carried out on the Cu-SHINs substrate covered by different thickness of SEI (Supplementary Fig. 5). It is seen that the growth of SEI does not weaken the electromagnetic field strength of the coupled plasmonic substrate, and the average EF of this configuration can still be as high as 10^{10}, which is similar to that of bare integrated substrate. This is because that the SEI components, such as LiF, Li_2O, LiOH, etc., absorb light weakly and their refractive indexes are very close to that of the electrolyte[1]. It may be necessary also to note that the thickness of SEIs on Li metal anodes typically ranges from several nanometers to tens of nanometers, which would not completely encapsulate or warp up a ~60 nm diameter Au@SiO_2 nanoparticle. Therefore, the enhanced Raman signals of SEI components arising from different depths of the inner and outer layer are achieved by hotspots located at the junctions of Cu island-to-Cu island, Cu island-to- SHIN and SHIN-to-SHIN, respectively, during SEI growth.

S5. The influence of Li deposition on the electromagnetic field distribution in the integrated Cu-SHINs substrate

In principle, Li is an s-electron metal and expected to provide strong LSPR effect. After Li deposition on the integrated Cu-SHINs substrate, the deposited Li would synergize with SHINs and become new SPR active sources to strengthen the plasmonic response and enhance Raman signals of SEI evolution. However, Li deposition has to proceed beneath a SEI, i.e. Li ion has to transport through SEI, which is then followed by nucleation and growth at metal/SEI interface. This could lead to two types of changes in the distribution of SHINs on the substrate upon Li growth, namely SHINs floating on and embedded in Li deposits, respectively, which has a significant impact on the electromagnetic field distribution. Note that SHINs are embedded in SEI in both cases as discussed above in Supplementary Note 2. To examine the electromagnetic-field distribution in the two cases, FEM simulations were performed and results are shown in Supplementary Fig. 6. It can be clearly seen that the electromagnetic field enhancement is weak to probe SEI when the SHINs are embedded in Li deposits, since most of hotspots are buried inside the Li deposits. On the contrary, nanoparticles floating on Li deposit can generate effective hotspots located at the junctions of Li-to-Li, Li-to-SHIN and SHIN-to-SHIN and thus still maintain the high enhancement factor of up to 10^{10}, which enables sensitive detection of Raman signals of SEI components.

S6. Chemical restructuring of SEI with participation of metallic Li

The SEIs formation can proceed chemically and/or electrochemically depending on reduction conditions and participation or not of active Li metal. On the Cu surface, SEIs are mainly formed by electrochemical reduction of anion and solvent molecules, and their exact composition are dictated by electrode potential during the potential-dependent formation processes. Taking electrolyte of LiTFSI/DME-DOL as an example, upon negative shifting of the potential, higher oxidation state species, such as $Li_2S_xO_y$, $ROCO_2Li$, $Li_2NSO_2CF_3$ would be formed and distribute non-uniformly in the vertical direction but mainly in the inner region. This lay basis for a primary Cu-SEI, which is less stable and more conductive though (Supplementary Figure 14). After initiation of Li OPD, high oxidation state species in both inner and outer regions would further decompose via additional chemical reactions under ample supply of active metallic Li. Consequently, the Cu-SEI undergoes restructuring to form Li-SEI, which facilitates formation of more stable low oxidation states products, such as inorganic LiF, Li_2S, and Li_3N; these low oxidation states products are less electron conductive, so that the thickness of the final SEI layer is reduced, compared to the primary SEI layer on Cu (Supplementary Figure 14). The combined chemistry and electrochemistry processes, especially the role of metallic Li, provide the possibility to actively control the formation of SEI and thus improve the quality of SEI.

Supplementary Figures

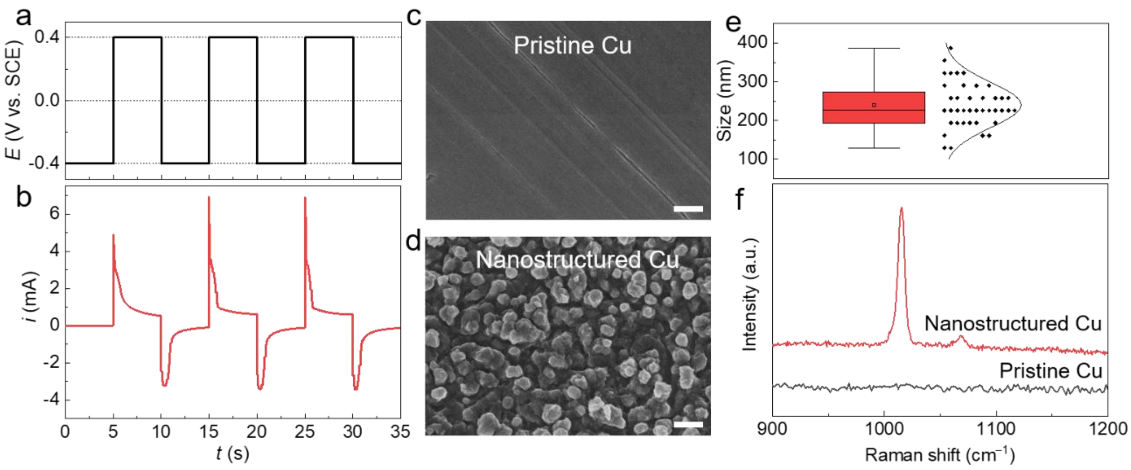

Supplementary Figure 1 | a,b, A procedure involving oxidation-reduction cycles accomplished by potential step from –0.4 V to 0.4 V to roughen the Cu electrode **(a)** and the corresponding *i*–*t* curves of Cu electrode following the electrochemical roughening produce **(b)**. **c,d,** The morphologies of Cu surface before **(c)** and after **(d)** electrochemical roughening. Scale bar: 1 μm. **e,** Size distribution of Cu nanoshperes on the Cu substrate. **f,** Raman spectra of the absorbed pyridine on different Cu substrates to test the effective SERS activation of the nanostructured Cu surface.

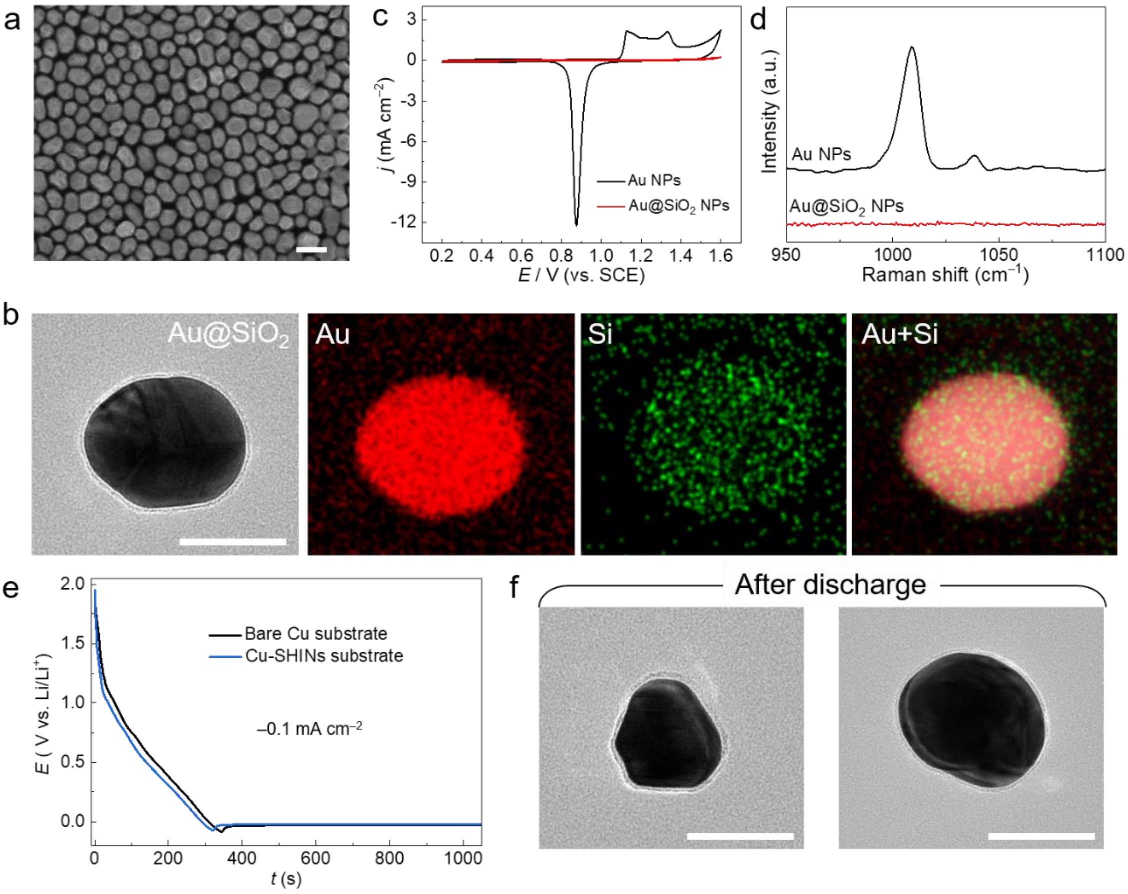

Supplementary Figure 2 | a, SEM image of $Au@SiO_2$ core-shell nanoparticles. Scale bar: 100 nm. **b,** TEM image and element maps of an $Au@SiO_2$ nanoparticle. Scale bar, 50 nm. **c,** Cyclic voltammograms of Au and $Au@SiO_2$ nanoparticles on glassy carbon electrodes in 0.5 M H_2SO_4 solution. Scan rate: 50 mV s^{-1}. **d,** Raman spectra of the absorbed pyridine from bare Au nanoparticles and pinhole-free $Au@SiO_2$ nanoparticles on Si wafer. **e,** Potential profiles of galvanostatic Li deposition on bare Cu and Cu-SHINs substrates, respectively, at −0.1 mA cm^{-2}. **f,** TEM images $Au@SiO_2$ nanoparticles after discharge. Scale bar, 50 nm.

We used cyclic voltammetry to check that the synthesized $Au@SiO_2$ core-shell nanoparticles with ultra-thin shells are really pinhole-free. As shown in Supplementary Fig. 2b, the black curve reveals typical feature of bare Au nanoparticles with a characteristic reduction peak of Au surface oxide at about 0.9 V, whereas the red curve shows the feature of $Au@SiO_2$ nanoparticles without the characteristic reduction peak, indicating the pinholes-free character of the ultra-thin SiO_2 layer. Furthermore, the electrode with $Au@SiO_2$ nanoparticles have already displayed very small charging/discharging currents of the electrochemical double layer

capacitance, which also indicates the inert surface electrochemical property of the thin SiO_2 shell. In addition to the electrochemical examination, we further used SERS method with pyridine as typical probing molecule for more sensitively checking whether the SiO_2 shell are pinhole-free. As shown in Supplementary Fig. 2c, there are no signals related to pyridine from Au@SiO_2 nanoparticles on Si wafer, which confirms the compactness of the ultra-thin SiO_2 shell coated on Au core nanoparticles. Additionally, we should mention that SiO_2 shell may have a potential risk of lithiation but highly depends on its size, crystallinity, morphology, and oxygen content. To inspect whether the SiO_2 shell in our system goes through the lithiation process, we conducted TEM characterization for discharged Au@SiO_2 nanoparticles. Specifically, we first performed galvanostatic discharge measurement on Cu-SHIN substrate in DOL-based electrolyte (Supplementary Fig. 2e). Then, the discharged Cu-SHIN substrate was washed with DME to remove residual electrolyte and put into DME solution for 1 h of ultrasonic cleanout, and finally the fallen Au@SiO2 SHINs were collected for TEM imaging. As shown in Supplementary Fig. 2f, the thickness of SiO_2 shell are virtually unchanged after discharge, compared to the initial SiO_2 shell with thickness of ~2 nm, and no volumetric strain induced by lithiation can be detected. The above-presented experiments suggest that the SiO_2 shell in our system does not undergo the lithiation process (or if it takes place, it is too inconspicuous to interfere with the experiments).

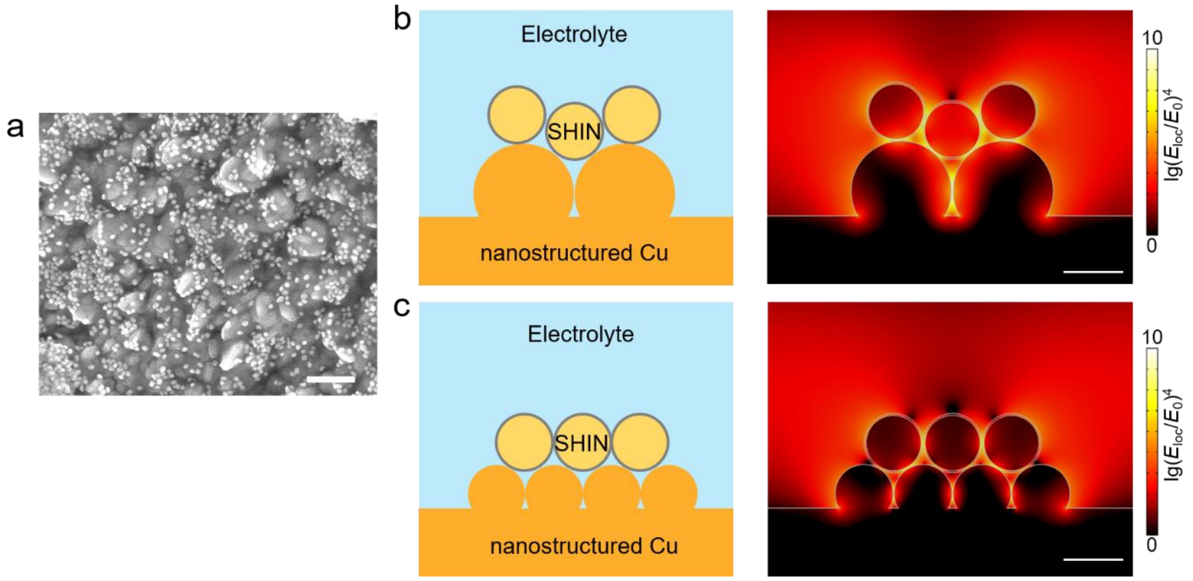

Supplementary Figure 3 | a, SEM image of SHINs spread initially on nanostructured Cu substrate. Scale bar, 600 nm. **b,c,** Schematic illustrations of the integrated Cu-SHINs substrate with larger **(b)** and smaller **(c)** Cu islands, respectively, along with FEM simulations of the corresponding Raman enhancement distribution. The refractive index of the electrolyte is 1.4. E_{loc} and E_0 represent the localized electric field and the incident electric field, respectively. Scale bar: 60 nm.

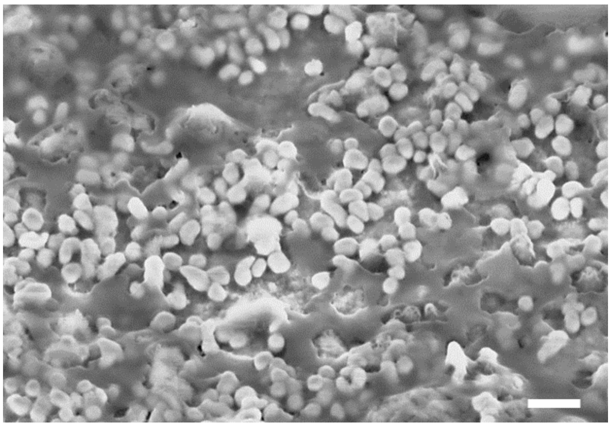

Supplementary Figure 4 | SEM image of SHINs distribution after Li deposition. Scale bar, 200 nm.

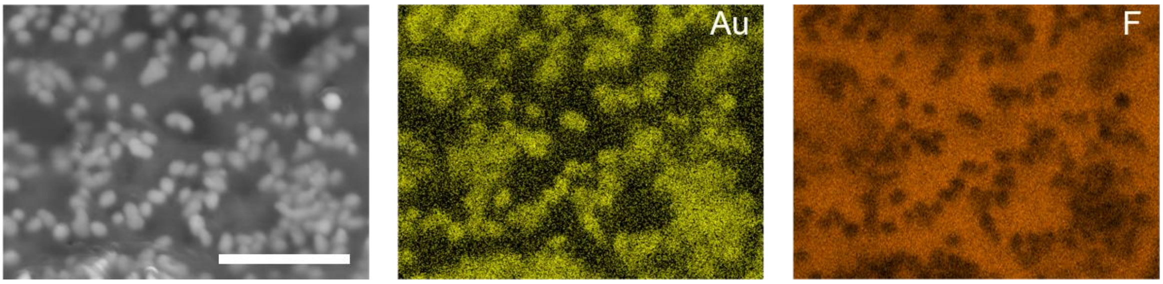

Supplementary Figure 5 | SEM image and elemental maps of Au@SiO_2 nanoparticles partially embedded in SEI formed on Cu electrode in the electrolyte of LiTFSI in DME-DOL.

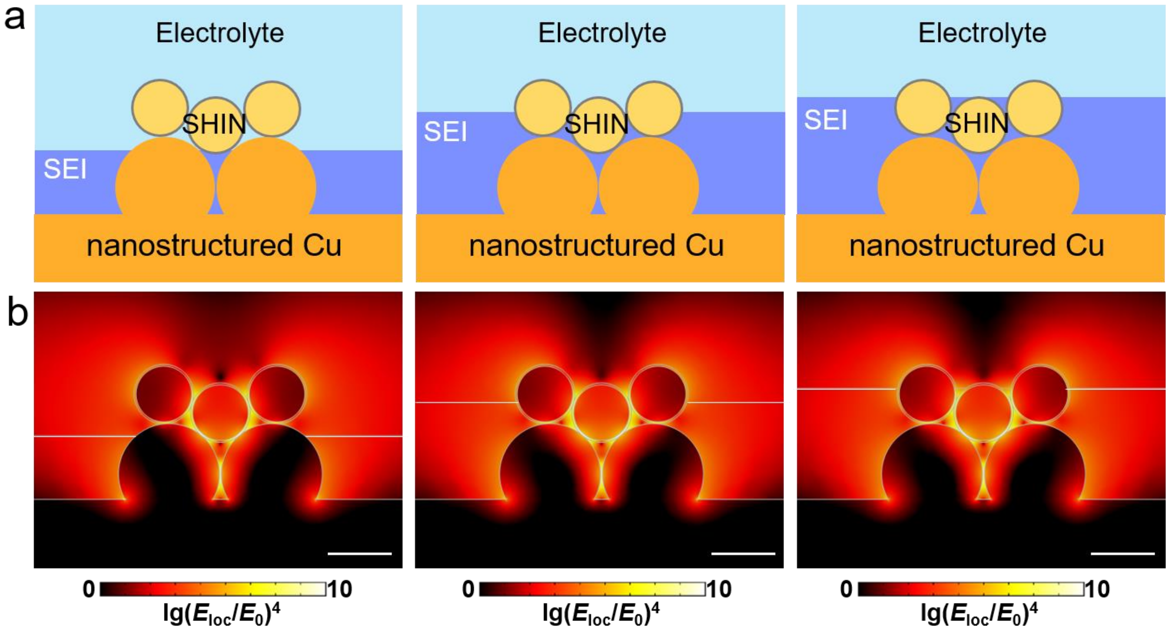

Supplementary Figure 6 | a, Schematic illustration of the integrated Cu-SHINs substrate covered by different thickness of SEI. **b,** The FEM simulations of the Raman enhancement distribution of the corresponding situations. The refractive index of the electrolyte and SEI are 1.4 and 1.6, respectively. E_{loc} and E_0 represent the localized electric field and the incident electric field, respectively. Scale bar: 60 nm.

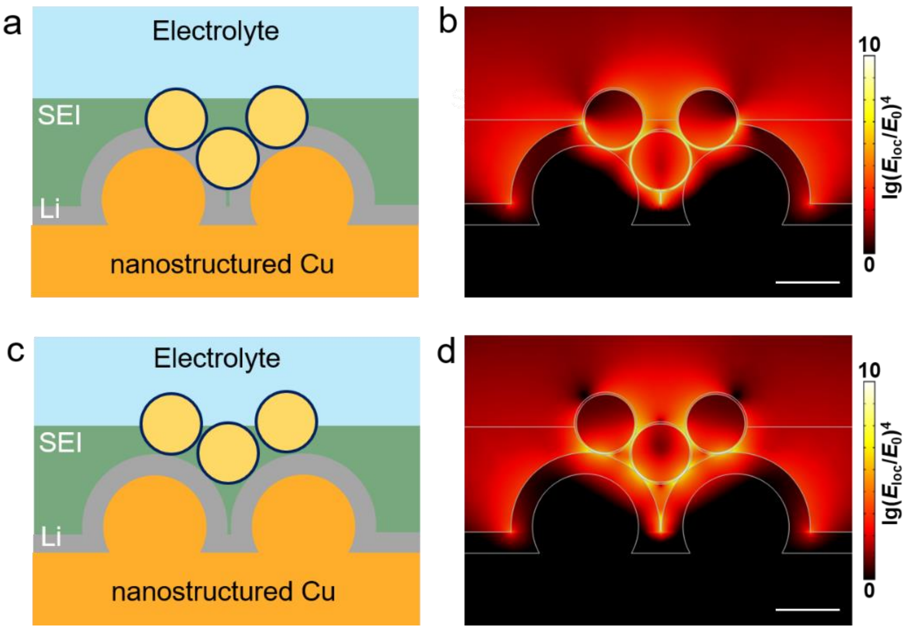

Supplementary Figure 7 | a, Schematic illustration of the SHINs embedded in deposited Li and SEI on the nanostructured Cu substrate. **b,** Finite-element simulations of the corresponding Raman enhancement distribution of configuration in **a**. Scale bar: 60 nm. **c,** Schematic illustration of the SHINs floating on deposited Li but embedded in SEI on the nanostructured Cu substrate. **d,** Finite-element simulations of the corresponding Raman enhancement distribution of configuration in **c**. Scale bar: 60 nm. For all the simulations, the refractive indexes of the electrolyte and SEI are 1.4 and 1.6, respectively. E_{loc} and E_0 represent the localized electric field and the incident electric field, respectively.

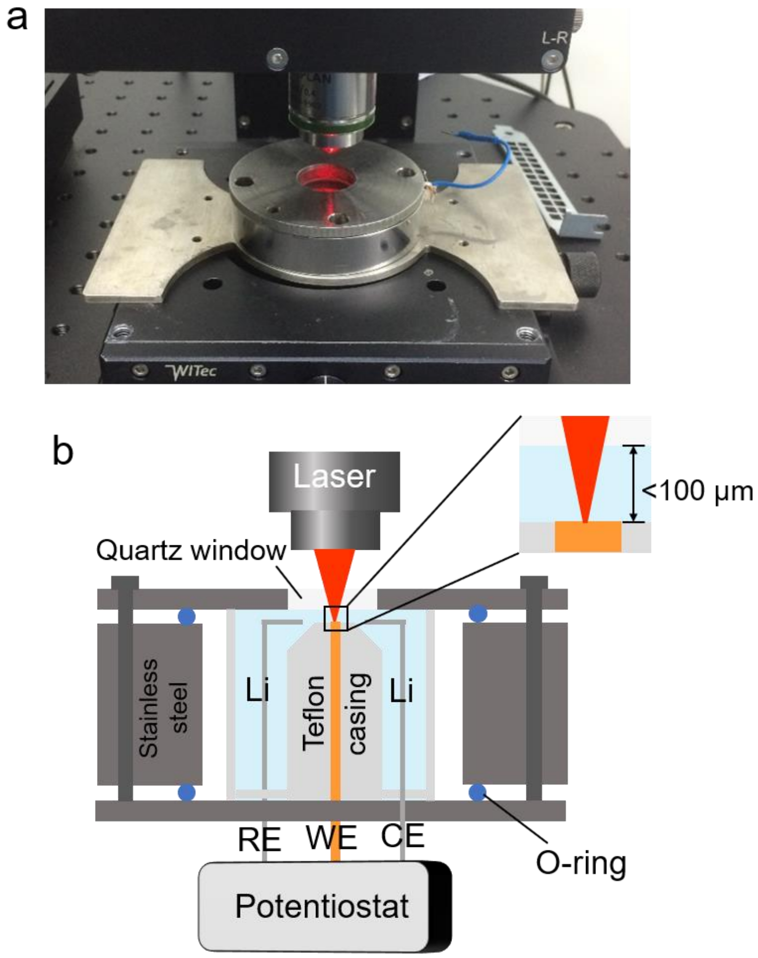

Supplementary Figure 8 | a, Photograph of the three-electrode Raman cell. **b,** Schematic of the Raman experimental setup.

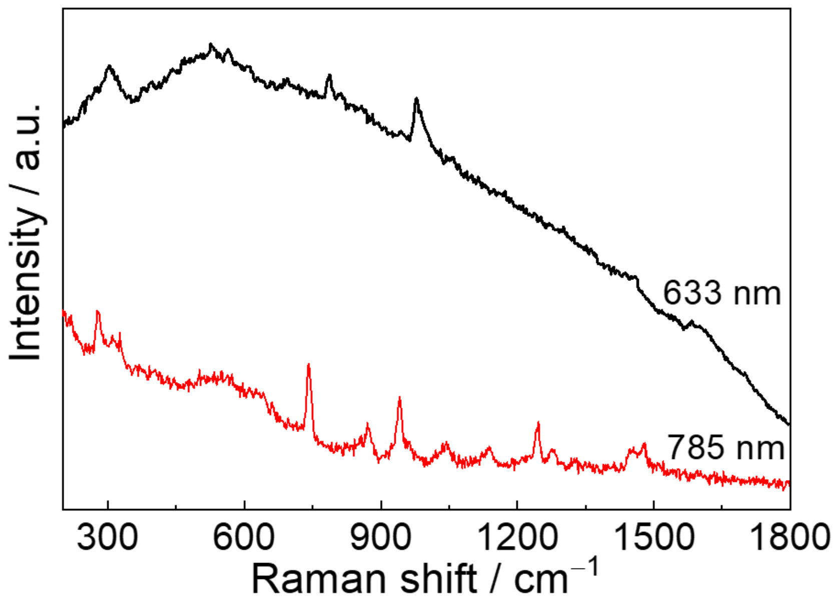

Supplementary Figure 9 | Raman spectra of 1M LiTFSI/DME-DOL electrolyte on integrated Cu-SHINs substrate under different excitation laser.

Measurement using a 785 nm excitation allow minimizing the strong fluorescence background of the electrolyte and recovery of the electrolyte Raman signature.

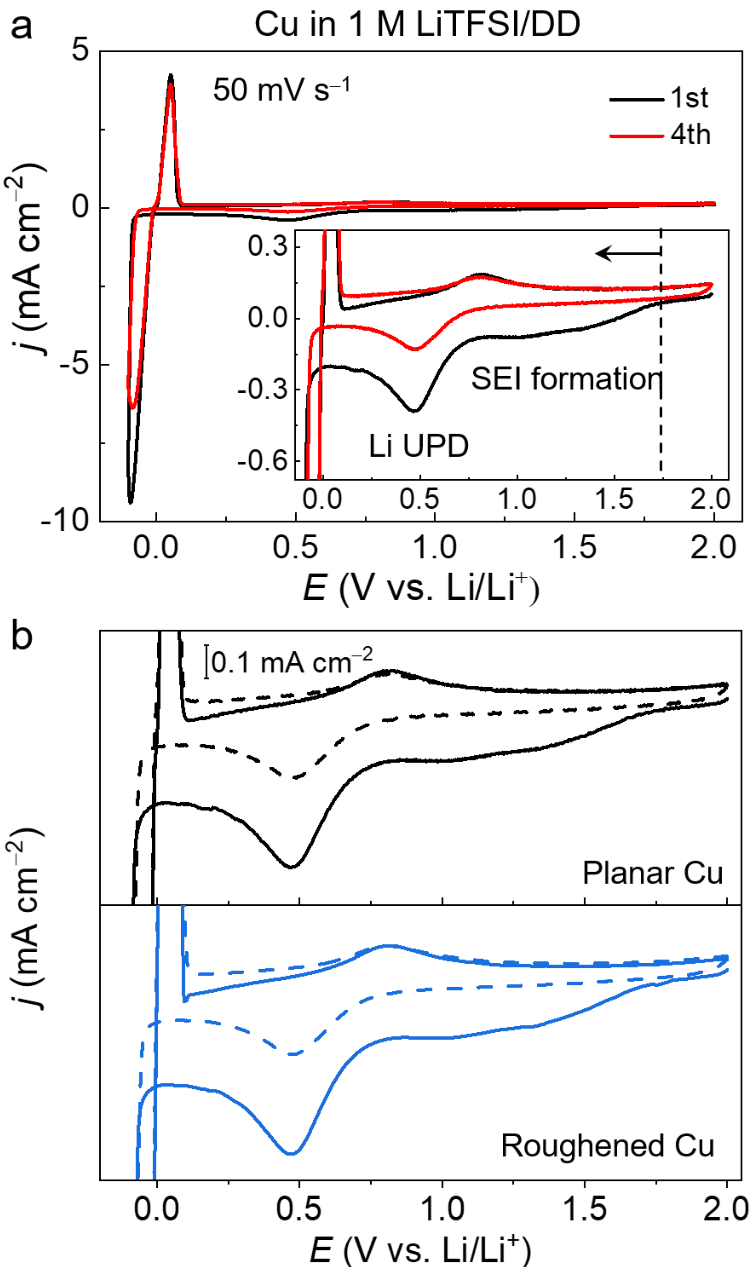

Supplementary Figure 10 | a, Cyclic voltammograms (CVs) of a Cu disk electrode in electrolyte of 1 M LiTFSI/DME-DOL (V/V, 1/1). Scan rate: 50 mV s^{-1}. **b,** Comparison of CVs of planar Cu and nanostructured Cu. The surface area of the nanostructured Cu is ca. 2 times of the planar Cu.

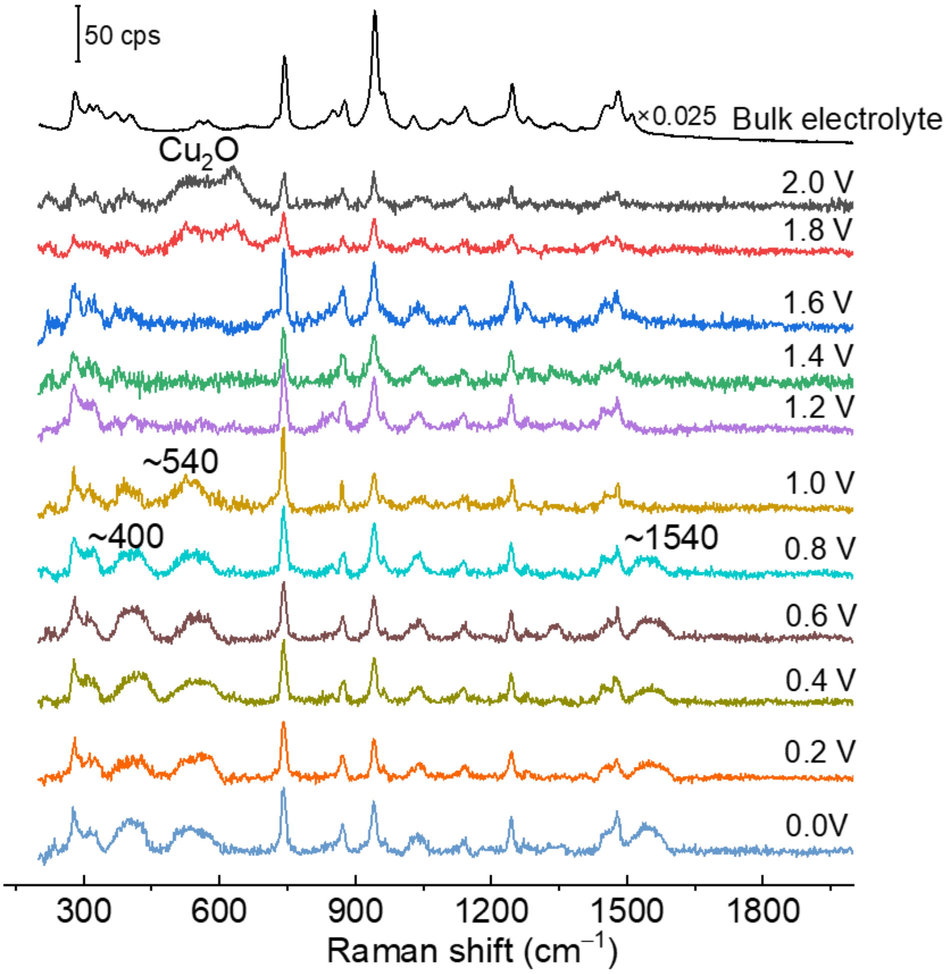

Supplementary Figure 11 | in-situ Raman spectra for the formation and evolution of SEI on bare nanostructured Cu substrate before Li overpotential deposition. Detailed band assignments are provided in Supplementary Table 1.

As shown in Supplementary Figure 10, other than the signals from electrolyte, two overlapping Raman bands at around 512 and 612 cm^{-1} from Cu_2O are visible at OCP, which are unavoidably formed during electrochemical roughening, but would gradually disappear upon decreasing the potential. The appearance of Cu_2O bands indicates that the obtained SERS signals are reliably from the electrode surface. More negative than 1.6 V, the Raman signals are amplified due to the enhancement of LSPR effect of Cu after removal of the oxide layer. It is noted that the intensity of the band at 743 cm^{-1} associated with TFSI anion vibration (S–N and C–S stretching, CF_3 bending) is stronger than that of the band at 942 cm^{-1} corresponding to DOL vibration (C–O and C–C stretching) on the electrode surface, which is opposite to the situation in bulk

electrolyte. This is not surprising since the anion concentration would increase and interaction of anion with electrode surface would become stronger even at OCP that is very likely positive of the potential of zero charge (PZC) of such a system, where the surface electron density is low. However, the intensity-to-intensity ratio of the two bands remain almost constant with negative shifting of potential. The reasons may be that the SEI formation affect the charge state of the electrode surface during negative potential excursion, which requires further investigation. In any case, the inversion of intensity-to-intensity ratio of the two prominent bands at 743 and 942 cm^{-1} is another indication of the obtained Raman signals from electrode surface.

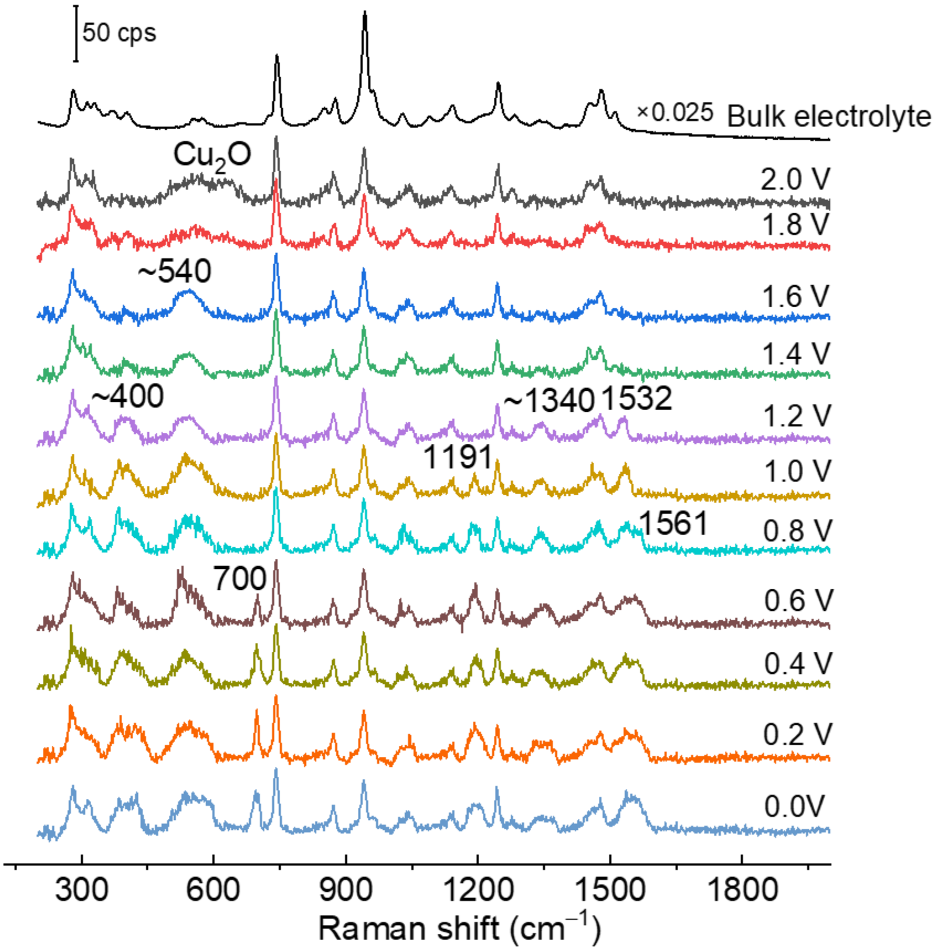

Supplementary Figure 12 | in-situ Raman spectra for the formation and evolution of SEI on integrated Cu-SHINs substrate before Li overpotential deposition. Detailed band assignments are provided in Supplementary Table 2.

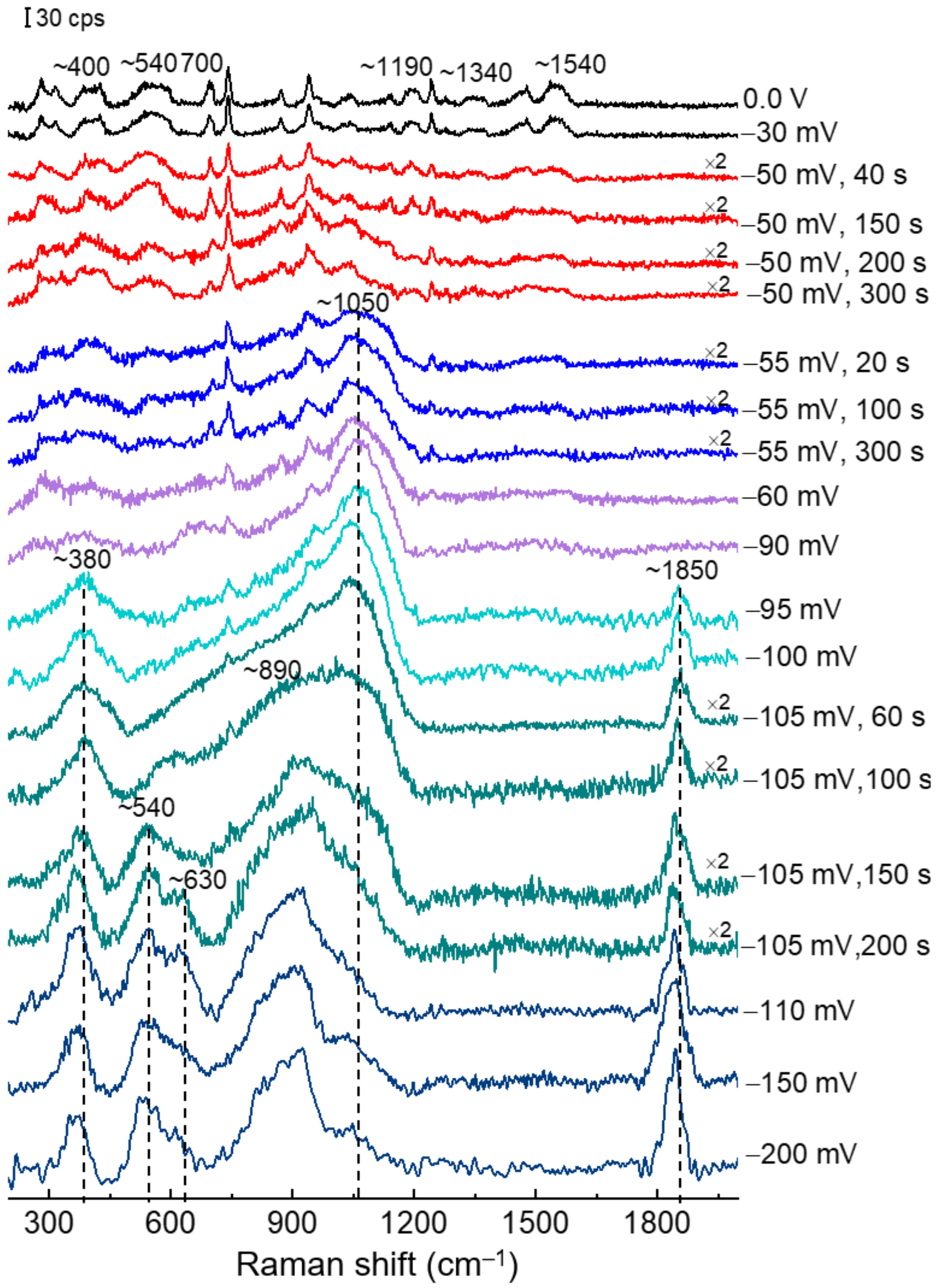

Supplementary Figure 13 | in-situ Raman spectra for the evolution of SEI on integrated Cu-SHINs substrate after Li overpotential deposition with precise control of applied potential and time. Detailed band assignments are provided in Supplementary Tables 2 and 3.

Supplementary Fig. 13 shows an in-situ Raman measurements for SEI evolution conducted in real-time with precise control of applied potential. It is clear that the broad band at ~1050 cm^{-1} emerges first and gradually intensifies after Li OPD, which indicates species of $Li_2S_xO_y$ and ROLi are first generated from their higher oxidation state counterparts such as $Li_2NSO_2CF_3$ due

to the chemical reactions with metallic Li. As the potential become more negative and with extension of time, broad bands at low wavenumber emerge gradually, which are ascribed to the further decompostion of higher oxidation state species to lower oxidation state ones such as Li_2O, LiF, Li_2S and Li_3N with the particapaticon of metallic Li. These results further clartify the main role of metallic Li is to promote additional chemical reactions, which significantly alters the structure of the primary SEI on Cu, resulting in different-featured final SEIs on Li.

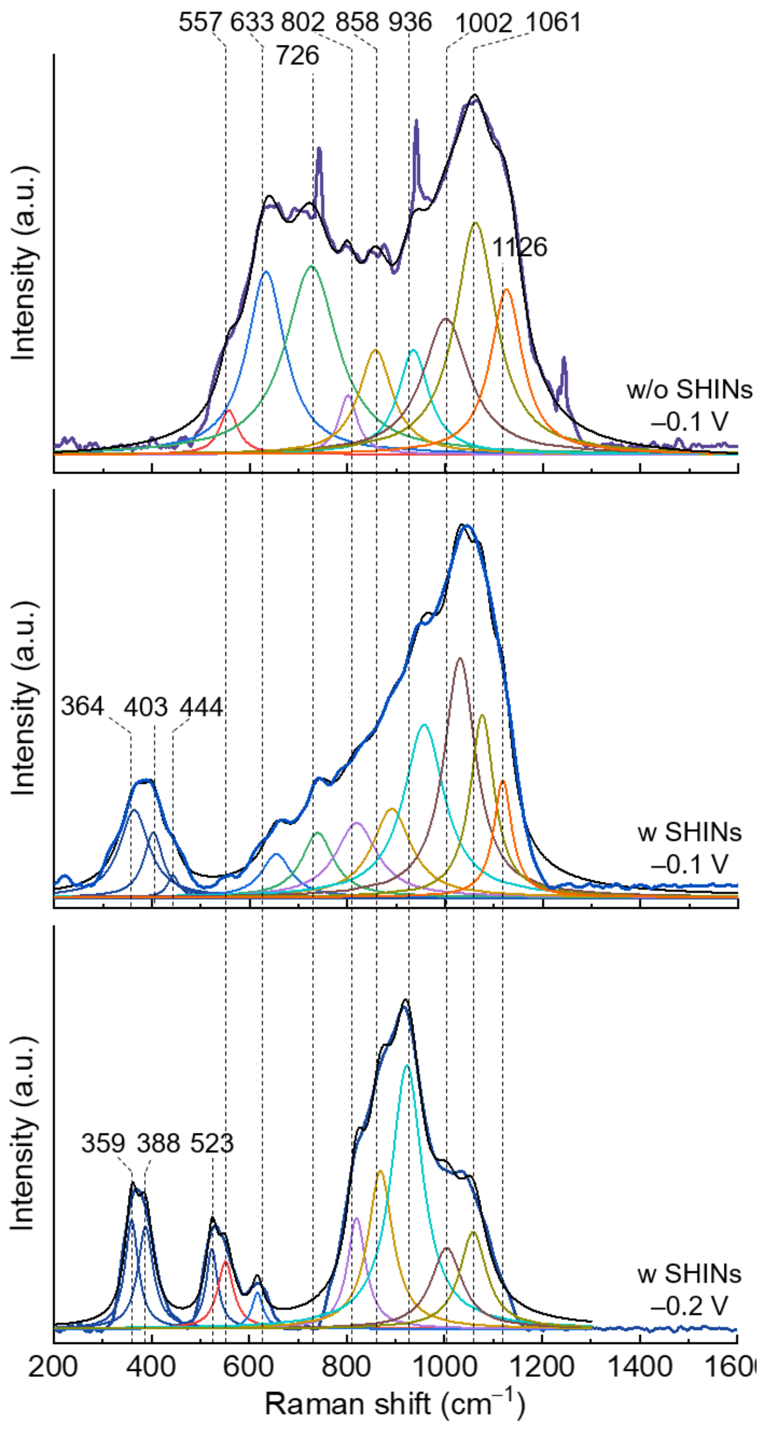

Supplementary Figure 14 | Lorentz fitting of the Raman spectra for the SEIs formed on bare nanostructured Cu substrate (upper) and integrated Cu-SHINs substrate (middle and lower) after Li overpotential deposition.

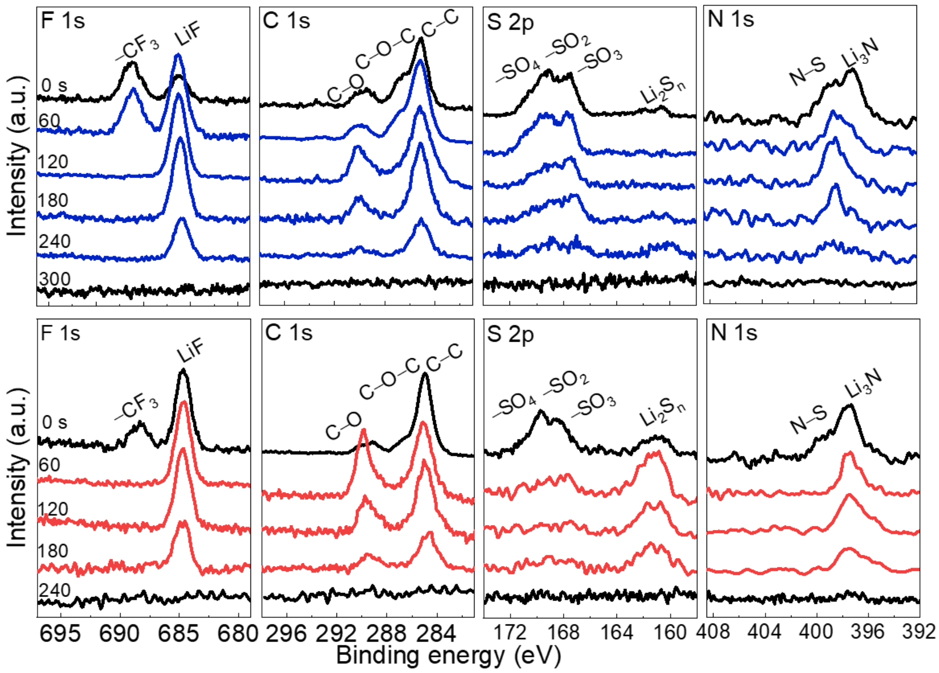

Supplementary Figure 15 | F 1s, C 1s, S 2p and N 1s XPS depth profiles of SEIs formed on Cu electrode before (upper) and after (lower) Li deposition.

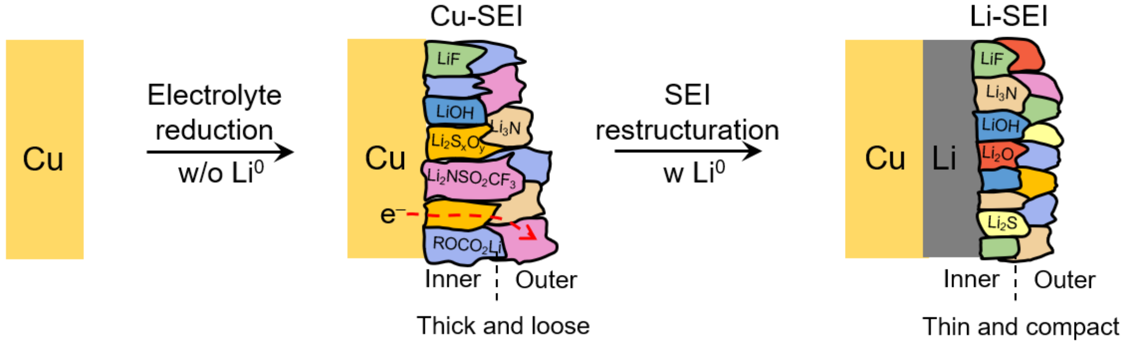

Supplementary Figure 16 | Schematic illustration of sequential formation of SEI on Cu and then Li that is in-situ deposited on Cu and SEI restructuration with participation of metallic Li.

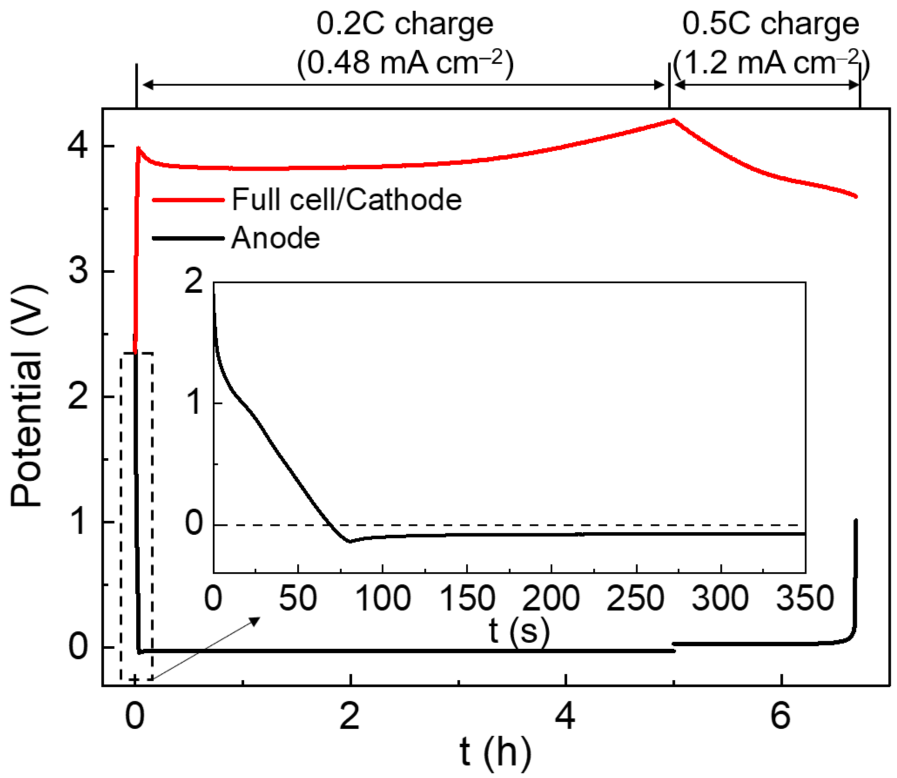

Supplementary Figure 17 | The voltage profiles of a typical anode-free cell with a Cu current collector as the anode and commercial NMC532 coated on Al foil as the cathode (15 mg cm^{-2}). The cell was cycled between 3.6 V and 4.5 V at C/5 rate for charge and C/2 rate for discharge. The electrolyte used was 0.6 M LiDFOB-0.6 M $LiBF_4$/DEC-FEC (V/V=2:1). The voltage profiles of the NMC532 cathode and Cu anode were monitored simultaneously using a three-electrode cell with a Li reference electrode.

It is clear that within the first approximately 80 s after the application of the current for charge, the potential on Cu anode is driven to close to but above 0 V, meaning that the electrolyte undergoes a rapid decomposition on the Cu surface to form primary SEI on Cu. Upon further charging to the potential below 0 V where Li overpotential deposition occurs, with distinct Li nucleation and growth, the formation of SEI proceeds further via both electrochemical and chemical routes.

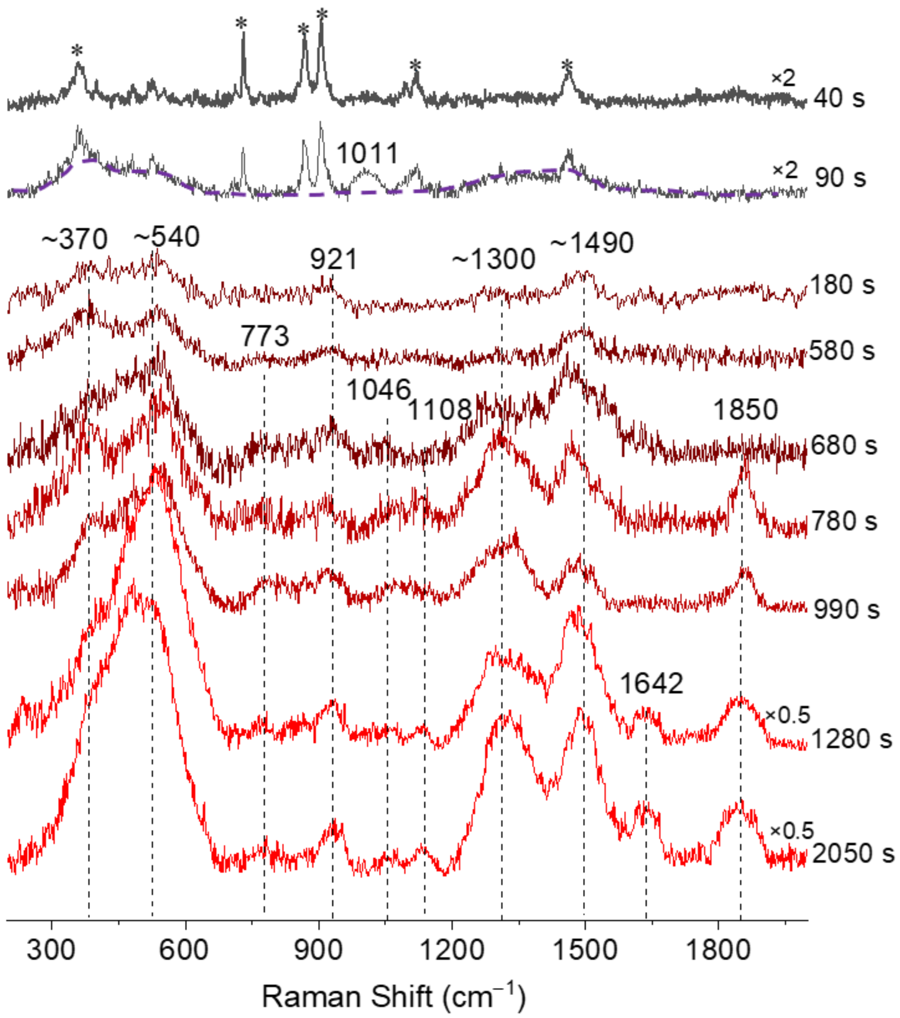

Supplementary Figure 18 | In-situ Raman spectra for the formation and evolution of SEI on integrated Cu-SHINs substrate during galvanostatic polarization at –0.48 mA cm^{-2}. Detailed band assignments are provided in Supplementary Table 4.

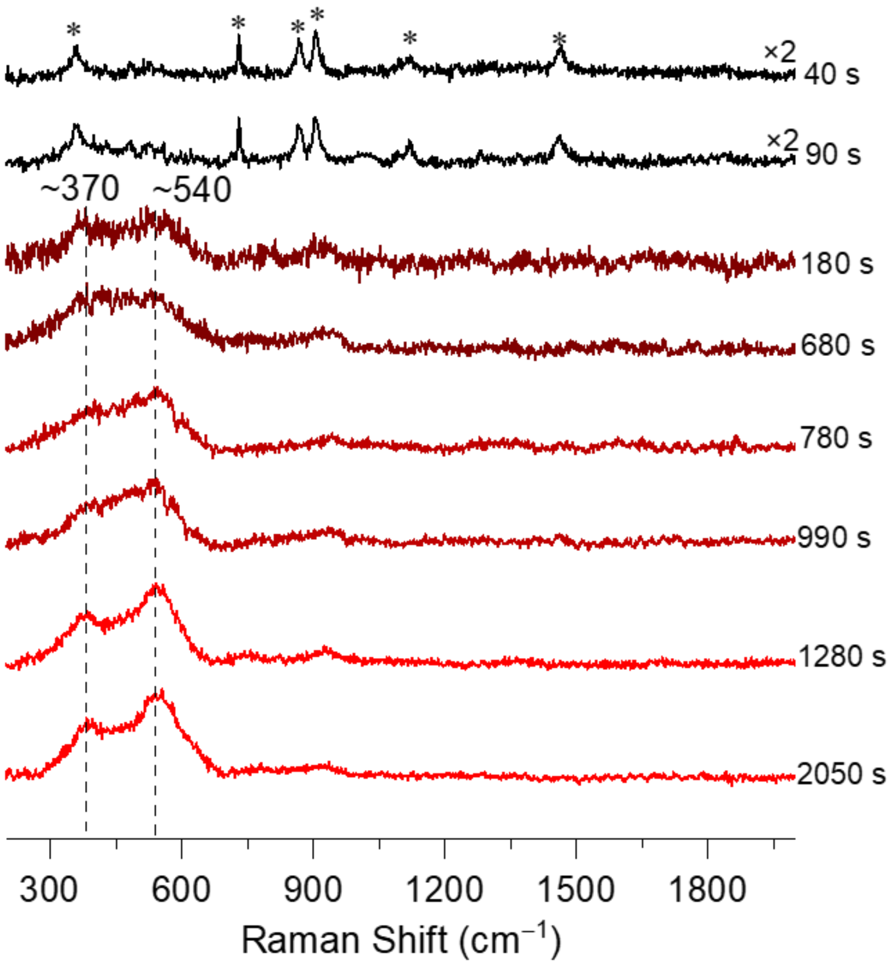

Supplementary Figure 19 | in-situ Raman spectra for the formation and evolution of SEI on bare nanostructured Cu substrate during galvanostatic polarization at –0.48 mA cm^{-2}. Detailed band assignments are provided in Supplementary Table 4.

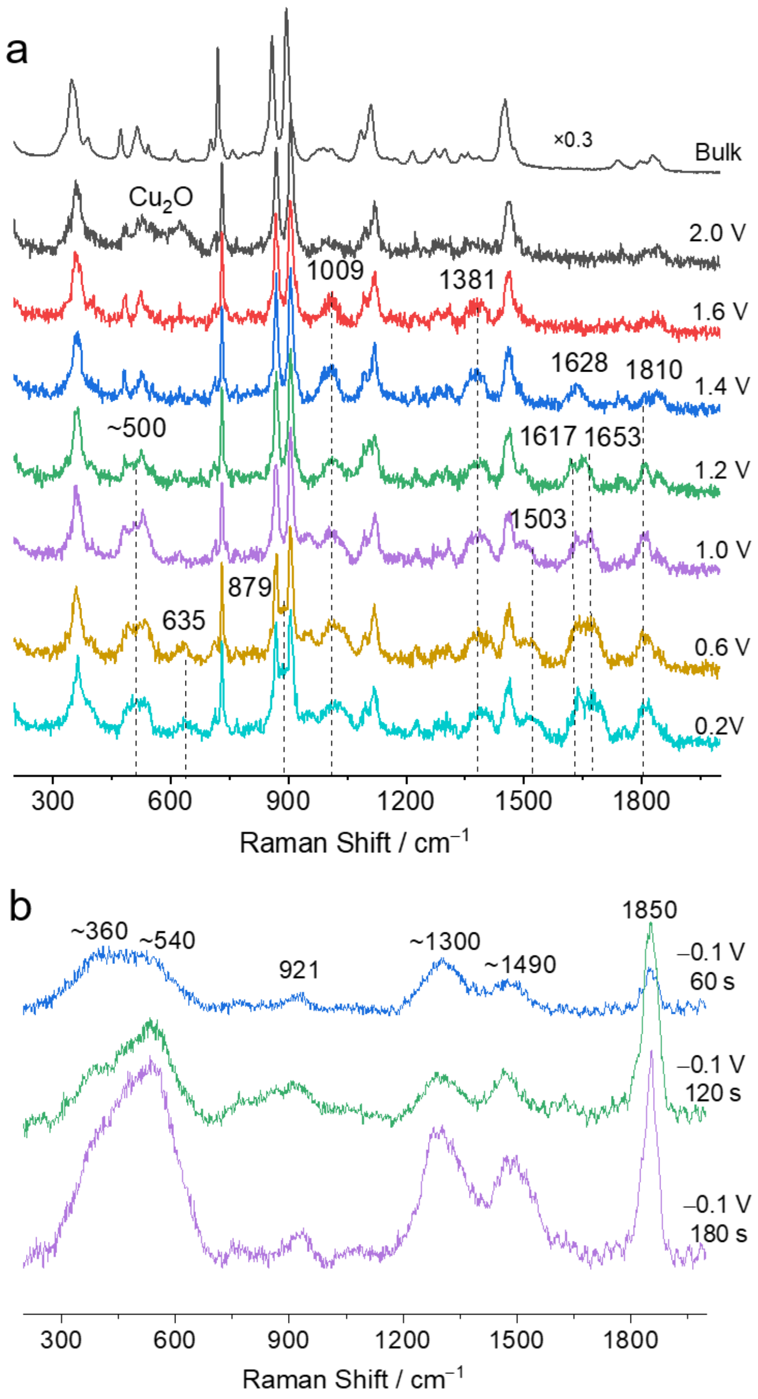

Supplementary Figure 20 | a,b, Potential-dependent Raman spectra for the formation and evolution of SEI on plasmonic Cu-SHINs coupled substrate before **(a)** and after **(b)** Li overpotential deposition. Detailed band assignments are provided in Supplementary Table 5.

To investigate the influence of primary Cu-SEI on the follow-up Li-SEI, we further performed potential-dependent Raman measurements on the Cu-SHINs coupled plasmonic substrate in the same electrolyte system as shown in Supplementary Fig. 19. Prior to Li OPD, several individual

bands appear during the negative potential excursion, whose vibrational assignment and principal chemical components are summarized in Supplementary Table 5. These bands are mainly from organic B-containing species as well as CO_2/CO_3 species, again confirming the organic feature of Cu-SEI in this case. Once Li OPD occurs, the broad bands associated with Li-SEI show up immediately, and their frequency and intensity are similar to those on the spectra in Fig. 3a in the main text. Especially, the band at 1850 cm^{-1} attributed to the symmetric stretching of C≡C bond from Li_2C_2 appears to be much strongly related to the one in Fig. 3a in the main text. It is noteworthy that there exist two different insights in the literature works regarding the mechanism for the appearance of Li_2C_2 species: Naudin *et al.* suggested the appearance of Li_2C_2 as a result of local degradation of organic species by laser-induced heating processes[5]; while Schmitz and co-workers believed that the band is an intrinsic species from SEI[6]. In either case, the presence of this band indicates that the SEI contains significant amounts of organic components. It is likely that the longer Cu-SEI proceeding, the more high-oxidation state organic components in final Li-SEI, especially in the outer layer.

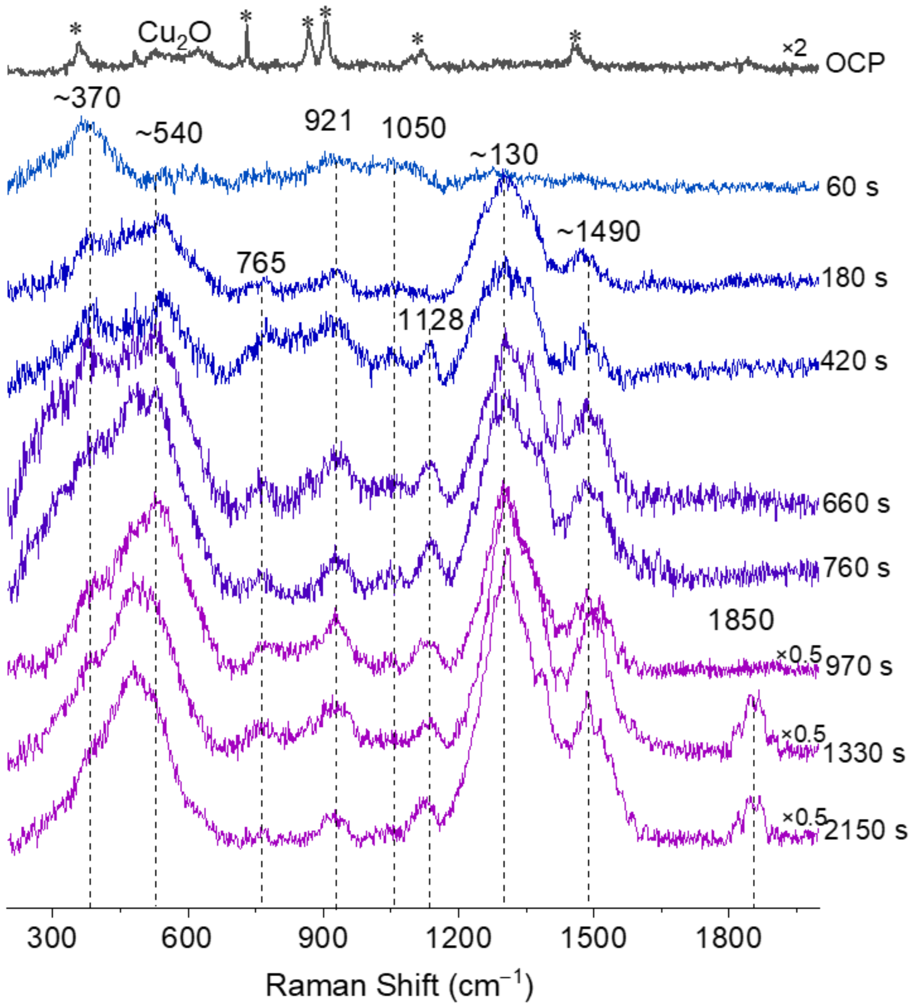

Supplementary Figure 21 | in-situ Raman spectra for the formation and evolution of SEI on integrated Cu-SHINs substrate during potentiostaic-galvanostatic polarization, including a potentiostatic step (–0.1 V, 100 s) to the region where concurrent Li OPD and electrolyte reduction takes place, and a follow-up galvanostatic plating (–0.48 mA cm^{-2}) during which concurrent Li OPD and electrolyte reduction further occurs. Detailed band assignments are provided in Supplementary Table 4.

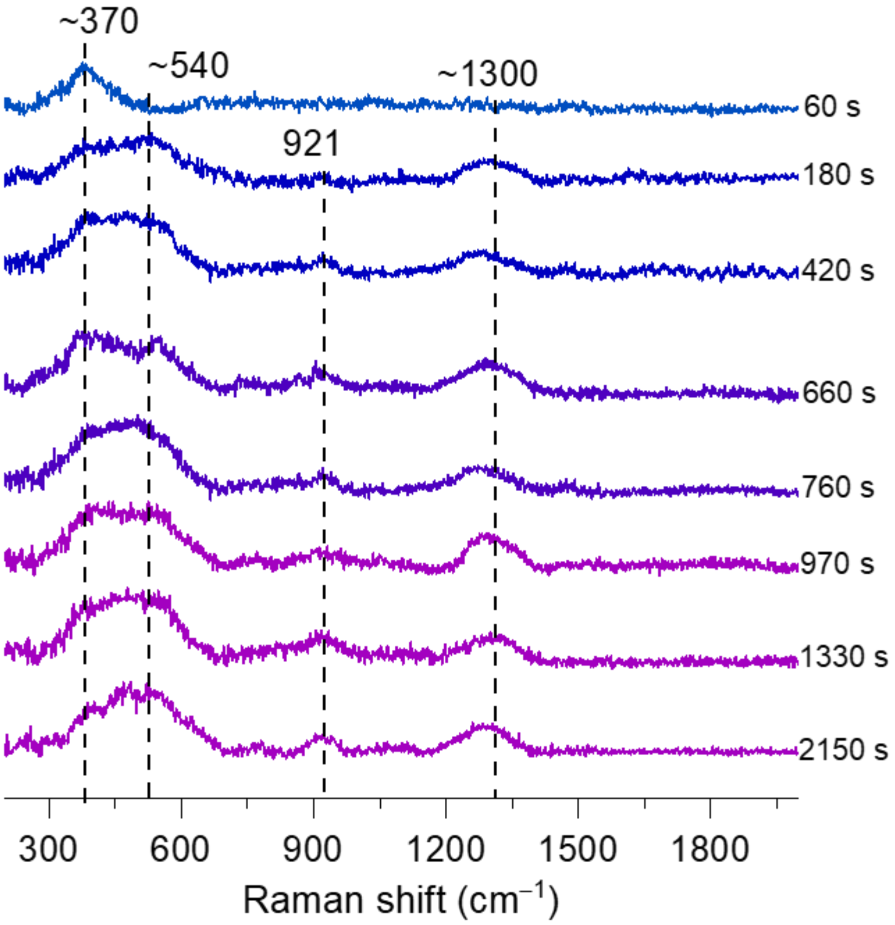

Supplementary Figure 22 | in-situ Raman spectra for the formation and evolution of SEI on bare nanostructured Cu substrate during potentiostaic-galvanostatic polarization, including a potentiostatic step (–0.1 V, 100 s) to the region where concurrent Li OPD and electrolyte reduction takes place, and a follow-up galvanostatic plating (–0.48 mA cm^{-2}) during which concurrent Li OPD and electrolyte reduction further occurs. Detailed band assignments are provided in Supplementary Table 4.

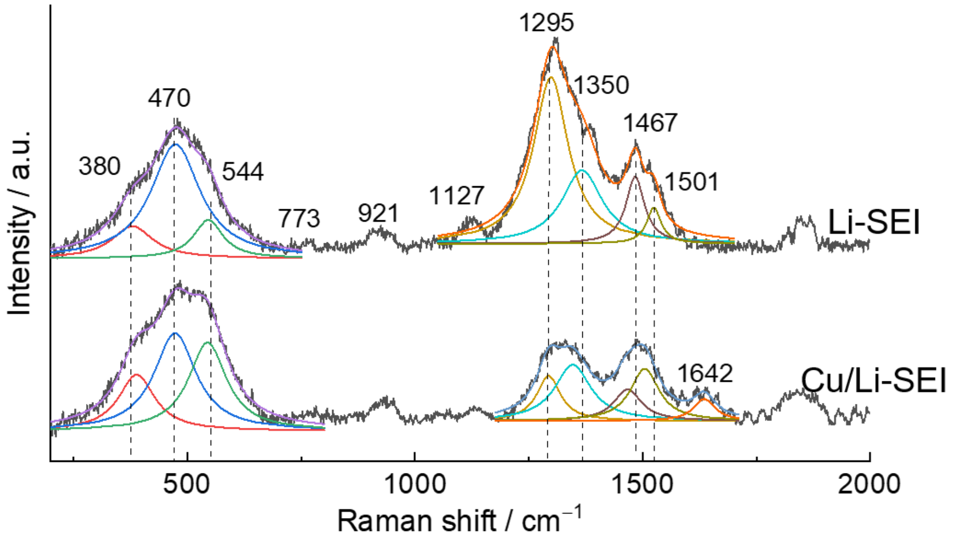

Supplementary Figure 23 | Lorentz fitting of the Raman spectra for the SEIs formed in normal galvanostatic formation route (lower) and potentiostatic-galvanostatic formation route (upper). Detailed band assignments are provided in Supplementary Table 4.

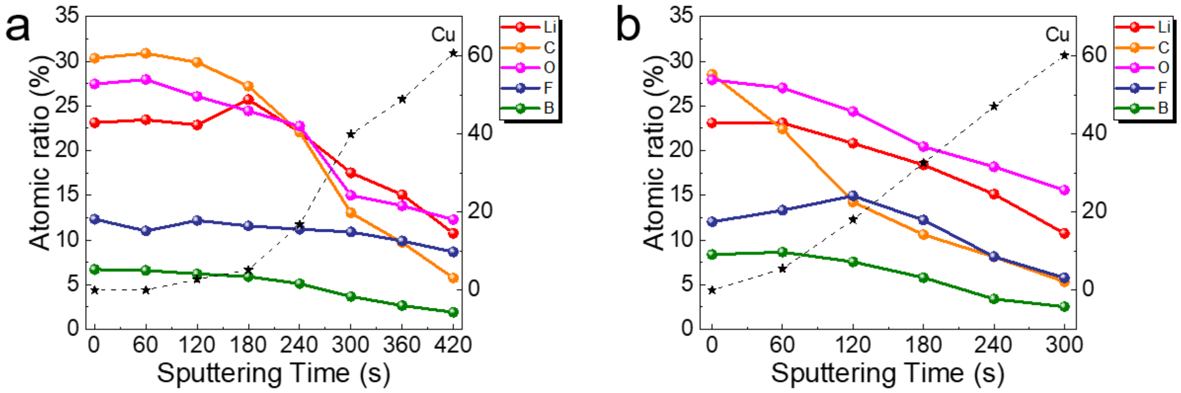

Supplementary Figure 24 | XPS depth profiles of the atomic concentration of the SEIs formed by normal galvanostatic formation route **(a)** and potentiostatic-galvanostatic formation route **(b)** in electrolyte of LiDFOB-$LiBF_4$/DEC-FEC.

It is clear seen that the thickness of SEI formed by potentiostatic-galvanostatic formation route is thinner (25 nm) than that of SEI formed by normal galvanostatic formation route (35 nm). This indicates that the formation of SEI with direct participation of metallic Li can promote the formation of low oxidation state products and thus reduce the thickness of SEI.

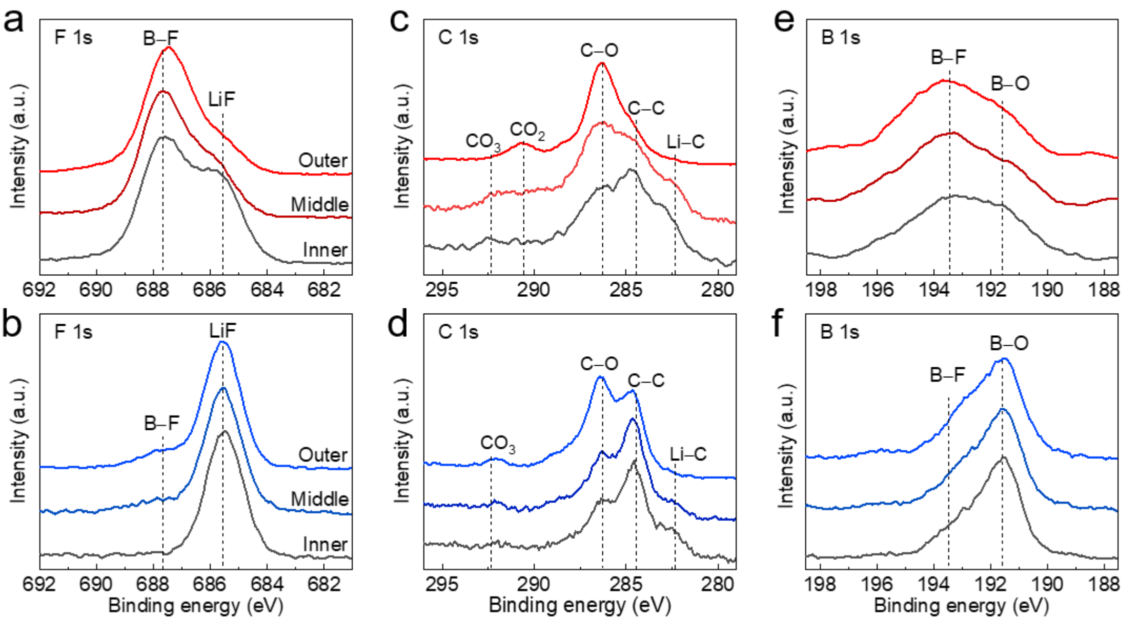

Supplementary Figure 25 | a–f, F 1s **(a,b)**, C 1s **(c,d)** and B 1s **(e,f)** XPS spectra for sequentially formed SEI **(a,c,e)** and directly formed SEI **(b,d,f)**, respectively.

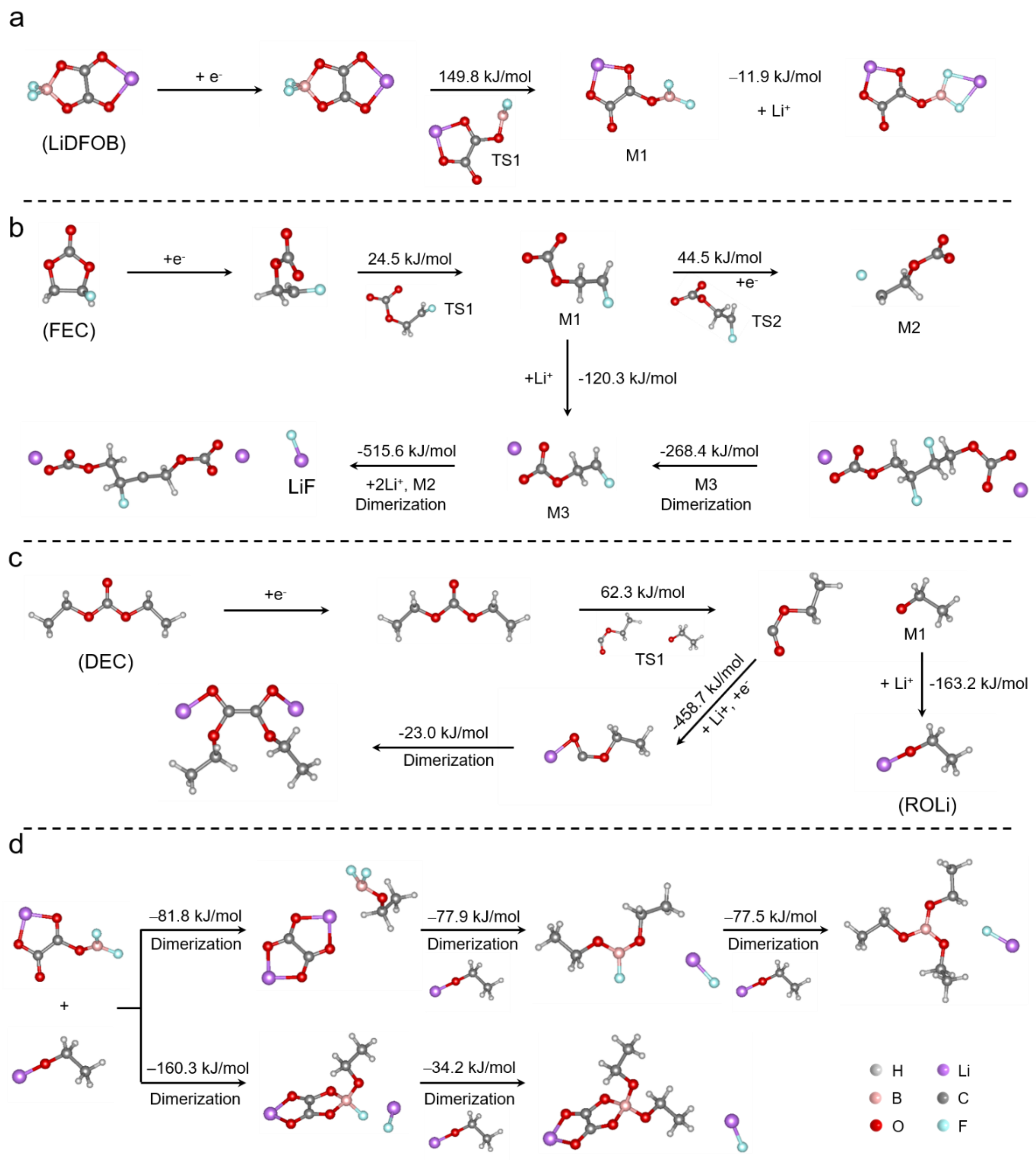

Supplementary Figure 26 | a–c, Proposed reduction reaction of LiDFOB **(a)**, FEC **(b)** and DEC **(c)** on the negative electrode. **d,** Proposed reactions of the electron-deficient B-containing intermediates from DFOB anion decomposition with electron-rich lithium alkoxide.

Supplementary Fig. 26 show the proposed reaction mechanism for decomposition of LiDFOB and solvents, which can be electrochemically-driven and/or chemically-driven depending on whether it initiates on the current collector or reactive Li metal surface. For the sequential

formation route, the reduction of DFOB anion initiates on the Cu surface and undergoes a partial ring-opening reaction (Fig. 4i in the main text) in relatively high potential region, which induces the B–O band cleavage and thus generates electron-deficient difluoroborate species. Meanwhile, the reduction of DEC coupled with a ring-opening reduction of FEC (Supplementary Fig. 25) allows for the generation of electron-rich Li alkoxides (ROLi) or Li carbonates ($ROCO_2Li$), which can subsequently react with difluoroborate. For the direct formation route, DFOB anions and solvents prefer capturing more electrons from reactive Li metal, which are then used for ring-opening reaction and thus produce electron-deficient products as well as low-oxidation state species such as LiF. In particular, reactions and polymerizations of the electron-deficient difluoroborate species with electron-rich ROLi or $ROCO_2Li$ could be accelerated via fluorine/oxygen exchange reaction on boron with plenty supply of metallic Li, which is conducive to generation of more LiF as well as polymeric components that consist of B-containing species like $–B(OCH_2CH_2)_n–$ (Fig. 4j in the main text). It has been a general consensus that SEI bearing polymeric-like structure composed of oligomers incorporated with inorganic species are usually elastic and more facile for Li ion conduction, which are beneficial to suppressing dendrite growth for uniform Li deposition. The proposed mechanisms are consistent with our experimental observations and provide some insights into the advantages of direct formation route with participation of metallic Li in improving cell performance.

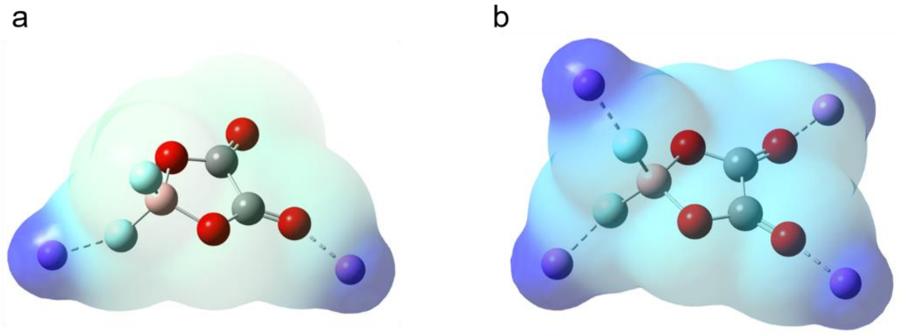

Supplementary Figure 27 | DFOB anion...Li$^+$ cation coordination. a, AGGs-I, DFOB coordinating to two Li-ions. **b,** AGGs-II, DFOB coordinating to four Li-ions.

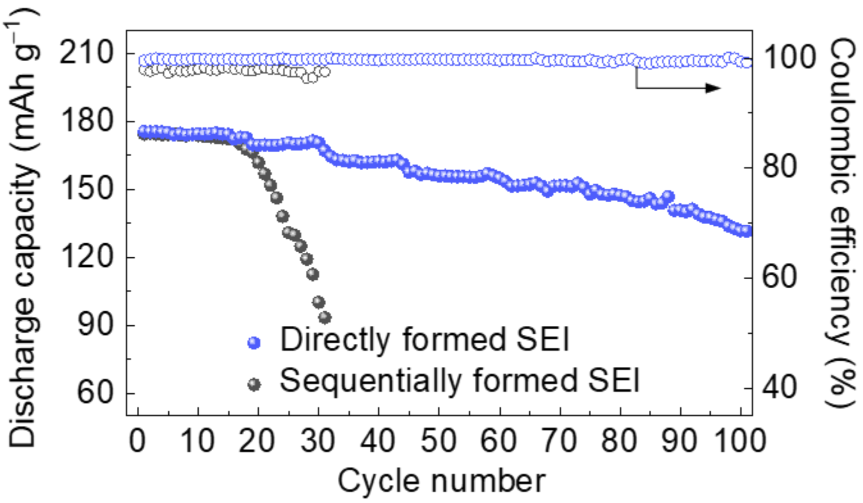

Supplementary Figure 28 | Electrochemical performances of anode-free electrodes covered by different SEIs. Specific discharge capacity versus cycle number and the corresponding Coulombic efficiency of the coin-type Cu‖NMC532 cells with Cu electrodes covered by different SEIs. These cells were cycled between 3.6 V and 4.5 V at the C/5 rate for charge and the C/2 rate for discharge. A 1C rate corresponds to a current density of 170 mA g^{-1} based on active NMC cathode material. Before the measurements, all the cells were subjected to two formation cycles at C/10 rate for both charge and discharge processes. It is demonstrated that a high average Coulombic efficiency (≈99.7%) as well as a high capacity retention 80% after 80 cycles can be achieved for Cu‖NMC532 cell with Cu electrode covered by directly formed SEI.

Supplementary Tables

Supplementary Table 1 | EF values of three types of hotspots located on the Cu-SHINs integrated substrate before and after SEI formation.

Hotspot in Cu-SHINs	EF values	
	Before SEI formation	After SEI formation
Hotspot-I	0.11×10^{10}	0.29×10^{10}
Hotspot-II	0.84×10^{9}	7.41×10^{9}
Hotspot-III	0.16×10^{9}	0.18×10^{9}

Supplementary Table 2 | Vibrational assignments of the main bands of Raman spectra for SEI formed on Cu electrode before Li overpotential deposition wth the electrolyte of LiTFSI/DME-DOL.

$\nu_{exp.}$ / cm^{-1}	$\nu_{ref.}$ / cm^{-1}	**Assignment**	**References***
~400	393	LiOH	Ref. 7
	404	LiF	Ref. 8
	404	$\omega SO_2/\nu C–F$	Ref. 9
~540	534	Li_2O	Ref. 10
	535	ROLi	Ref. 11
	541	$\nu C–F$	Ref. 9
700	700	Li_3N	Ref. 12
1191	1192	νCF_3	Ref. 9
~1340	1330–1350	νSO_2, $\nu_s SO_2–N$	Ref. 9
	1300–1400	$\delta C–H$ (ROLi)	Ref. 11
~1540	1500–1580	νCO_2	Ref. 11

δ = bending, ν = stretching, ω = wagging

Supplementary Table 3 | Vibrational assignments of the main bands of Raman spectra for SEI formed on Cu electrode after Li overpotential deposition with the electrolyte of LiTFSI/DME-DOL.

$\nu_{exp.}$ / cm^{-1}	$\nu_{ref.}$ / cm^{-1}	Assignment	References*
359	352<br>356	τSO_2<br>LiOH	Ref. 9<br>Ref. 13
364	363<br>367	δC–O–C<br>LiOH	Ref. 11<br>Ref. 7
388	389<br>380	LiF<br>Li_2S	Ref. 8<br>Ref. 14
403	~400<br>404<br>404	Li_3N<br>LiF<br>ωSO_2/νC–F	Ref. 15<br>Ref. 8<br>Ref. 9
444	435	ROLi	Ref. 16
523	529<br>519	Li_2O<br>LiOH	Ref. 17<br>Ref. 7
557	500–600	ROLi	Ref. 11
633	631<br>639	νS–O<br>LiF	Ref. 9<br>Ref. 18
726	~717<br>720<br>733	Li_3N<br>$ROCO_2Li$<br>νC–F	Ref. 15<br>Ref. 16<br>Ref. 9
802	800–900	νS–O/νC–O	Ref. 19
858	855<br>800–900	δCO_3<br>νS–O/νC–O	Ref. 20<br>Ref. 19
936	929<br>943	νC–O<br>νS–O	Ref. 16<br>Ref. 19
1002	1009<br>1000–1100	ROLi<br>νS–O	Ref. 16<br>Ref. 19
1061	1060<br>1063<br>1065<br>1000–1100	$ROCO_2Li$<br>νC–O–C<br>CO_3^{2-}<br>νS–O	Ref. 20<br>Ref. 11<br>Ref. 21<br>Ref. 19
1126	1125<br>1131	ROLi<br>νS–O	Ref. 11<br>Ref. 9

δ = bending, ν = stretching, τ = twisting

Supplementary Table 4 | Vibrational assignments of the main bands of Raman spectra for Li-SEI formed on Cu electrode by the normal galvanostatic route and potentiostatic-galvanostatic route with the electrolyte of LiDFOB-$LiBF_4$/DEC-FEC.

$\nu_{exp.}$ / cm^{-1}	$\nu_{ref.}$ / cm^{-1}	$\nu_{calc.}$ / cm^{-1}	Assignment	References
380	382	—	LiOH	Ref. 7
470	—	469	ν_{as}Li–O in $(RO)_2BC_2O_4Li$	—
	470	—	$(LiF)_n$	Ref. 18
	474	—	δO–C–O (DFOB)	Ref. 22
544	—	542	νLi–O in $RFOCO_2Li$ (FEC)	—
		545	νLi–O in ROLi (DEC)	
		538	νB–F/ρB–O in $ROBFC_2O_4Li$	
773	780	—	$LiBO_2$	Ref. 23
921	—	920	νB–F in $ROBFC_2O_4Li$	—
1011	950–1050	1005	νB–O/υC–O (DFOB)	Ref. 24
1127	1100–1200	—	$CHFCH_2O$ (FEC)	Ref. 25
1295	—	1299	ν_{as}O–B–O in $B(OCH_2CH_2)_3$	—
	—	1293	δCH_2 in $(RO)_2BC_2O_4Li$	—
	1298	—	δO–B–O	Ref. 26
1350	—	1348	ωCH_2 in ROLi/$ROCO_2Li$	—
	1359	—	$Li_2C_2O_4$ (DFOB)	Ref. 22
1467, 1511	—	1400–1500	νC–O in ROLi	—
	1400–1500	—	νC=O, Li_2CO_3	Ref. 25,27
1642	1650	—	$ROCO_2Li$ (FEC/DEC)	Ref. 28

δ = bending, *ν* = stretching, ρ = rocking, *ω* = wagging

Supplementary Table 5 | Vibrational assignments of the main bands of Raman spectra for Cu-SEI formed on Cu electrode by the galvanostatic route with the electrolyte of LiDFOB-$LiBF_4$/DEC-FEC.

$\nu_{exp.}$ / cm^{-1}	$\nu_{ref.}$ / cm^{-1}	$\nu_{calc.}$ / cm^{-1}	Assignment	References*
~500	517	—	LiOH	Ref. 7
	500–600	500–600	ROCOOLi	Ref. 20
635	639	—	LiF	Ref. 18
879	872	875	ROCOOLi	Ref. 29
	870–890	—	δCO_3^{2-}	Ref. 20
1009	1009	1000	ROCOOLi	Ref. 27
	1010	1005	νB–O/νC–O	Ref. 24
1381	1375	1377	νB–O	Ref. 30
	1380	—	νC–O–O	Ref. 31
	1387	—	ROCOOLi	Ref. 29
1503	1497	1501	νB–O	Ref. 23
1617, 1628, 1653	1610–1680	1600	νC=O	Ref. 11
1810	~1800	—	$ROCO_2Li$ (FEC/DEC)	Ref. 32

δ = bending, *ν* = stretching

Supplementary References

1 Tang, S. *et al.* An electrochemical surface-enhanced Raman spectroscopic study on nanorod-structured lithium prepared by electrodeposition. *J. Raman Spectrosc.* **47**, 1017–1023 (2016).

2 Lin, D., Liu, Y. & Cui, Y. Reviving the lithium metal anode for high-energy batteries. *Nat. Nanotechnol.* **12**, 194–206 (2017).

3 Jin, S., Jiang, Y., Ji, H. & Yu, Y. Advanced 3D current collectors for lithium-based batteries. *Adv. Mater.* **30**, 1802014 (2018).

4 Zheng, J. *et al.* Regulating electrodeposition morphology of lithium: towards commercially relevant secondary Li metal batteries. *Chem. Soc. Rev.* **49**, 2701–2750 (2020).

5 Naudin, C. *et al.* Characterization of the lithium surface by infrared and Raman spectroscopies. *J. Power Sources* **124**, 518–525 (2003).

6 Schmitz, R. *et al.* SEI investigations on copper electrodes after lithium plating with Raman spectroscopy and mass spectrometry. *J. Power Sources* **233**, 110–114 (2013).

7 Li, G., Li, H., Mo, Y., Chen, L. & Huang, X. Further identification to the SEI film on Ag electrode in lithium batteries by surface enhanced Raman scattering (SERS). *J. Power Sources* **104**, 190–194 (2002).

8 Liu, F., Wang, G., Cheng, G., Han, M. & Ma, J. Fine structures of Raman spectra of LiF cluster-based nanofilms. *Solid State Commun.* **99**, 369–373 (1996).

9 Rey, I. *et al.* Spectroscopic and theoretical study of $(CF_3SO_2)_2N^-$ ($TFSI^-$) and $(CF_3SO_2)_2NH$ (HTFSI). *J. Phys. Chem. A* **102**, 3249–3258 (1998).

10 Hy, S., Felix, F., Rick, J., Su, W. N. & Hwang, B. J. Direct in situ observation of Li_2O evolution on Li-rich high-capacity cathode material, $Li[Ni_xLi_{(1-2x)/3}Mn_{(2-x)/3}]O_2$ ($0 \leq x \leq 0.5$). *J. Am. Chem. Soc.* **136**, 999–1007 (2014).

11 Cabo-Fernandez, L., Bresser, D., Braga, F., Passerini, S. & Hardwick, L. J. In-situ electrochemical SHINERS investigation of SEI composition on carbon-coated $Zn_{0.9}Fe_{0.1}O$ anode for lithium-ion batteries. *Batteries & Supercaps* **2**, 168–177 (2019).

12 Chandrasekhar, H. R., Bhattacharya, G., Migoni, R. & Bilz, H. Infrared and Raman spectra and lattice dynamics of the superionic conductor Li_3N. *Phys. Rev. B* **17**, 884–893 (1978).

13 Hy, S. *et al.* In situ surface enhanced Raman spectroscopic studies of solid electrolyte interphase formation in lithium ion battery electrodes. *J. Power Sources* **256**, 324–328 (2014).

14 Cheng, Q. *et al.* Full Dissolution of the whole lithium sulfide family (Li_2S_8 to Li_2S) in a safe eutectic solvent for rechargeable lithium–sulfur batteries. *Angew. Chem. Int. Ed.* **58**, 5557–5561 (2019).

15 Chandrasekhar, H. R., Bhattacharya, G., Migoni, R. & Bilz, H. Phonon spectra and lattice dynamics of lithium nitride. *Solid State Commun.* **22**, 681–684 (1977).

16 Matsuta, S., Asada, T. & Kitaura, K. Vibrational assignments of lithium alkyl carbonate and lithium alkoxide in the infrared spectra An ab initio MO study. *J. Electrochem. Soc.* **147**, 1695–1702 (2000).

17 Gittleson, F. S., Ryu, W. H. & Taylor, A. D. Operando observation of the gold–electrolyte interface in Li–O_2 batteries. *ACS Appl. Mater. Interfaces* **6**, 19017–19025 (2014).

18 Oschetzki, D. & Rauhut, G. Pushing the limits in accurate vibrational structure calculations: anharmonic frequencies of lithium fluoride clusters $(LiF)_n$, $n = 2–10$. *Phys. Chem. Chem. Phys.* **16**, 16426–16435 (2014).

19 Aurbach, D. *et al.* On the surface chemical aspects of very high energy density, rechargeable Li–sulfur batteries. *J. Electrochem. Soc.* **156**, A694–A702 (2009).

20 Verma, P., Maire, P. & Novák, P. A review of the features and analyses of the solid electrolyte interphase in Li-ion batteries. *Electrochim. Acta* **55**, 6332–6341 (2010).

21 Pasierb, P., Komornicki, S., Rokita, M. & Rękas, M. Structural properties of Li_2CO_3–$BaCO_3$ system derived from IR and Raman spectroscopy. *J. Mol. Struct.* **596**, 151–156 (2001).
22 Edwards, H. G. M. & Lewis, I. R. FT-Raman spectroscopic studies of metal oxalates and their mixtures. *Spectrochim. Acta, Part A* **50**, 1891–1898 (1994).
23 Rulmont, A. & Almou, M. Vibrational spectra of metaborates with infinite chain structure: LiBO2, CaB2O4, SrB2O4. *Spectrochim. Acta, Part A* **45**, 603–610 (1989).
24 Kang, S. H., Abraham, D. P., Xiao, A. & Lucht, B. L. Investigating the solid electrolyte interphase using binder-free graphite electrodes. *J. Power Sources* **175**, 526–532 (2008).
25 Tsubouchi, S. *et al.* Spectroscopic analysis of surface layers in close contact with edge Plane graphite negative-electrodes. *J. Electrochem. Soc.* **160**, A575–A580 (2013).
26 Karuppasamy, K. *et al.* An enhanced electrochemical and cycling properties of novel boronic Ionic liquid based ternary gel polymer electrolytes for rechargeable Li/$LiCoO_2$ cells. *Sci. Rep.* **7**, 11103 (2017).
27 Parimalam, B. S. & Lucht, B. L. Reduction Reactions of Electrolyte Salts for Lithium Ion Batteries: $LiPF_6$, $LiBF_4$, LiDFOB, LiBOB, and LiTFSI. *J. Electrochem. Soc.* **165**, A251–A255 (2018).
28 Hong, S., Choo, M.-H., Kwon, Y. H., Kim, J. Y. & Song, S.-W. Mechanisms for stable solid electrolyte interphase formation and improved cycling stability of tin-based battery anode in fluoroethylene carbonate-containing electrolyte. *Adv. Mater. interfaces* **3,** 1600172 (2016).
29 Gajan, A. *et al.* Solid electrolyte interphase instability in operating lithium-ion batteries unraveled by enhanced-Raman spectroscopy. *ACS Energy Lett.* **6**, 1757–1763 (2021).
30 Chen, J. *et al.* Sodium-difluoro(oxalato)borate (NaDFOB): a new electrolyte salt for Na-ion batteries. *Chem. Commun.* **51**, 9809–9812 (2015).
31 Schedlbauer, T. *et al.* Lithium difluoro(oxalato)borate: A promising salt for lithium metal based secondary batteries? *Electrochim. Acta* **92**, 102–107 (2013).
32 Nie, M. & Lucht, B. L. Role of lithium salt on solid electrolyte interface (SEI) formation and structure in lithium ion batteries. *J. Electrochem. Soc.* **161**, A1001–A1006 (2014).
